# Differences in the Effect of Dopamine on the Phototransduction Between Lampreys and Jawed Vertebrates

**DOI:** 10.3390/ijms27031435

**Published:** 2026-01-31

**Authors:** Darya A. Nikolaeva, Alexander Yu. Rotov, Irina Yu. Morina, Michael L. Firsov, Irina V. Romanova, Luba A. Astakhova

**Affiliations:** Sechenov Institute of Evolutionary Physiology and Biochemistry, RAS, 194223 Saint-Petersburg, Russia

**Keywords:** dopamine, dopamine receptors, photoreceptors, lamprey, phototransduction, cAMP

## Abstract

Dopamine is one of the most important neurotransmitters for regulating retinal function and adjusting vision to the diurnal cycle. It exerts its regulatory effects, in part, through the cAMP pathway. Previous studies have demonstrated that dopamine affects phototransduction in amphibian rods, and that elevated intracellular levels of cAMP modulate the function of vertebrate rods and cones. Lamprey, the most primitive vertebrate, could be valuable for studying the evolution of dopamine regulatory loops in the retina. We examined whether the photoresponse properties of long (cone-like) and short (rod-like) photoreceptors in the river lamprey could be regulated by dopamine via the cAMP pathway. Using suction pipette recording, we demonstrated that forskolin-induced elevation of cAMP has no effect on long or short photoreceptors. At the same time, dopamine modifies the photoresponse properties of long, but not short, photoreceptors at high, potentially non-physiological concentrations. Immunohistochemical analysis of the lamprey retina revealed the expression of both D1 and D2 dopamine receptors in lamprey photoreceptors; however, their distribution differs from jawed vertebrates. Taken together, our results suggest that, in lampreys, dopamine does not regulate photoreceptor sensitivity to light in the circadian rhythm, but, rather, adjusts other retinal functions based on widespread distribution of its receptors.

## 1. Introduction

The vertebrate retina adapts its functions to the day/night cycle to regulate vision. Neurotransmitters are vital for this process, with dopamine being one of the most significant [1,2]. Dopamine in the retina is produced and released almost exclusively by a specialized type of cells known as dopaminergic amacrine cells [3]. The release of dopamine in response to light is driven by several photoreceptor pathways, in which rods play a major role, but cones and melanopsin-expressing intrinsically photosensitive retinal ganglion cells (ipRGCs) also contribute to the activation of these dopaminergic amacrine cells under different lighting conditions [4]. The level of dopamine in the retina fluctuates cyclically, increasing during the day and decreasing at night. Dopamine in the retina of vertebrates regulates the communication between different types of retinal cells through gap junctions, forming both homotypic and heterotypic coupling [5,6,7,8], and affects the transmission of signals via glutamate and GABA receptors in retinal neurons [9,10]. It also acts on numerous other cellular targets (reviewed in [11,12]). With regard to photoreceptor cells, it has been demonstrated that dopamine is involved in the signal transduction between photoreceptors via gap junctions [5,13,14]; the regulation of synaptic transmission between rods, cones, and horizontal cells [15,16]; hyperpolarization-activated currents in rod photoreceptors [17,18]; retinomotor movements [19,20]; and the phagocytosis of outer segment membrane discs [21,22]. We have previously shown that dopamine and dopamine receptor agonists affect the functioning of the phototransduction cascade in isolated amphibian rods, modulating their sensitivity and response kinetics [23]. Dopamine decreases rod sensitivity by reducing the activation rate of the cascade and, to a lesser extent, by speeding up its deactivation. Furthermore, as previously demonstrated, elevated intracellular levels of cAMP significantly increase the light sensitivity of amphibian rods [24] and markedly modulate the response and dark current recovery in fish cones [25,26,27]. As intracellular cAMP is modulated via dopamine receptors [28], these regulatory loops could serve as the basis for dopamine-mediated signaling when adapting the phototransduction cascade to diurnal variation in ambient light.

Extracellular dopamine acts on retinal neurons through specific receptor proteins. Based on their ability to modulate cAMP production, dopamine receptors can be divided into two classes: D1-type and D2-type [29,30,31]. Activation of these receptors triggers a variety of intracellular events, which may or may not be related to changes in cAMP concentration within a cell. Most major retinal cell types appear to express dopamine receptors. D1-type receptors have been identified in horizontal, bipolar, ganglion, amacrine, and pigment epithelial cells [32,33,34,35]. In the retinas of vertebrates, D2-like receptors are expressed by both classes of photoreceptors—rods and cones [20,36,37], and mainly D4-receptors. Also, D2-like receptors are expressed by horizontal cells [38] and by bipolar, ganglion, and amacrine cells [37,39]. All of this knowledge about the functions of dopamine, its signaling, and the distribution of dopamine receptors in the retina has been obtained from different classes of vertebrates whose retinas and cell types are contemporary from an evolutionary standpoint. However, it would be interesting to shed light on the evolution of dopamine loops in the retina. In this context, the lamprey’s retina could be a useful model. Lampreys belong to the jawless class of vertebrates and are the most primitive currently existing vertebrates; their ancestors diverged from other vertebrate species over 500 million years ago [40,41]. Nevertheless, lampreys have well-designed, camera-type eyes with an orderly, layered retina and neuron types that are conserved between jawless and jawed lineages. These include photoreceptors, bipolar, horizontal, amacrine, and ganglion cells [42,43]. The lamprey retina is duplex and has distinct rod- and cone-like photoreceptors [44,45]. Nevertheless, the retinas of jawless and higher vertebrates exhibit notable differences (reviewed in [42]). In particular, lamprey rods are morphologically similar to cones; the outer segments of both consist of invaginating lamellae formed by the plasma membrane. Additionally, in the lamprey retina, retinal ganglion cell bodies flank the inner plexiform layer (IPL), with their axons converging in the optic fiber layer (OFL) between the IPL and inner nuclear layer (INL).

The present study aimed to investigate whether the photoresponse properties of the long (cone-like) and short (rod-like) photoreceptors in the lamprey’s retina could be regulated by dopamine and changes in intracellular cAMP levels. We compared the results of this study with those obtained from the experiments on the effects of dopamine and forskolin on frog rods [24,27] and Carassius cones (this work, [25]). The rods of marsh frogs are typical twilight vision photoreceptors, exhibiting high sensitivity (they enable response to light stimuli measured in units of photons), and having large outer segments. They also perform very stably in experiments involving recordings from isolated rods [46]. Regarding Carassius cones, we worked with green-sensitive cones (one of the four photoreceptor spectral types found in this species’ retina) to study the effects of dopamine. These are typical photoreceptors of daytime vision. They have normal cone morphology (thin and relatively short outer segments), low sensitivity, and fast photoresponses. Based on our experience, they demonstrate acceptable stability in experiments involving the recording of isolated photoreceptors [46].

Dopamine is assumed to activate D4 receptors (which belong to the D2-like family) in both rods and cones of vertebrates, thereby reducing the cAMP pool (see [11] for a review). In our previous work on frog rods [23], however, we postulated that not all of the observed effects on the phototransduction cascade in this type of photoreceptor could be explained by the cAMP-mediated action of dopamine alone. The present study aimed to evaluate the potential effects of dopamine on lamprey photoreceptors. A separate series of experiments involving direct cAMP elevation using forskolin was designed to distinguish cAMP-dependent from cAMP-independent effects of dopamine.

The second part of the study focused on the immunohistochemical evaluation of dopamine receptor expression in the various layers and cell types of the lamprey’s retina, which remains unclear to our knowledge.

## 2. Results

### 2.1. Single-Cell Suction Pipette Recordings

The aim of this study was to investigate the potential role of dopamine and the cAMP signaling pathway in the phototransduction cascade in two types of photoreceptors, long and short, in the retina of the river lamprey. Given the distinct physiological characteristics of these photoreceptors—long photoreceptors exhibit fast photoresponses and relatively low light sensitivity, resembling the cones of higher vertebrates, while short photoreceptors display slow photoresponses and high light sensitivity, becoming saturated at low light levels and resembling the rods of higher vertebrates—one might expect different contributions from the dopamine- and cAMP-mediated regulatory loops.

#### 2.1.1. Control Experiments

In our experiments, we applied dopamine and forskolin to different types of photoreceptors over an extended period of time (≥30 min perfusion) using solutions containing the tested pharmacological agents for each cell. To account for potential changes in the main photoresponse parameters over time, we performed control experiments in which each photoreceptor type was kept in Ringer’s solution for an equivalent period. The photoresponses were recorded at 20 and 30 min time points in the control experiments following the protocol described in Section 4. These data were then used to extract “control” parameters for further statistical analysis.

#### 2.1.2. The Effects of Dopamine on the Photoresponses of Long and Short Photoreceptors in the River Lamprey

*Short photoreceptors*. Electrophysiological experiments were carried out according to the established protocol. The first set of photoresponses was recorded in Ringer’s solution. After a 20 and 30 min perfusion with a dopamine-containing solution (2.5, 50, or 250 μM), the responses were recorded again using the same light stimulation parameters. The tested sets of responses included the following: (1) response to a saturating flash—R_max_, (2) responses to a near quarter-saturating flash—R_1/4_, and (3) responses to a near half-saturating flash—R_1/2_. During data processing, R_1/4_ and R_1/2_ were normalized to the dark current value at the corresponding time point to enable comparison of parameters across experimental conditions. The normalized fractional responses were used to measure the photoreceptor sensitivity based on their amplitudes; they were also used to determine kinetic parameters. The amplitudes of R_max_ were used as a measure of dark current. All parameters are expressed as ratios of post- to pre-incubation values and compared to those obtained from corresponding control experiments.

**Figure 1:**
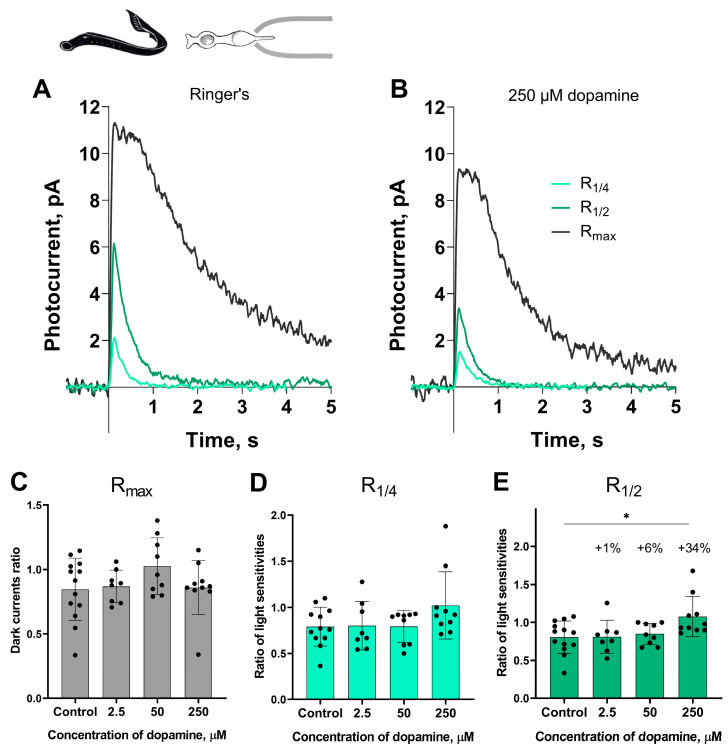
The effects of 2.5, 50, and 250 μM dopamine on lamprey short photoreceptors. A typical set of responses of lamprey short photoreceptors to saturating, nearly half-saturating, and quarter-saturating flashes in Ringer’s solution (**A**) and after 30 min of 250 μM dopamine exposure (**B**). Flash intensities are 1.03 × 10^4^ photons/μm^2^ per flash (saturating), 246 photons/μm^2^ per flash (near half-saturating), and 98 photons/μm^2^ per flash (near quarter-saturating); λ_max_ = 525 nm. (**C**) shows the effects of dopamine at the tested concentrations on the dark current, and (**D**,**E**) demonstrate dopamine effects on light sensitivity to near quarter-saturating and near half-saturating flashes, respectively. * *p* = 0.0145 (Dunnett’s multiple comparisons test).

Figure 1 shows a typical set of responses from lamprey short photoreceptors: R_max_, R_1/4_, R_1/2_ in Ringer’s solution and after 30 min of dopamine exposure. No significant impact was observed on the dark current (R_max_) or light sensitivity (R_1/4_) to near quarter-saturating flashes. However, we observed a small but statistically significant increase in sensitivity (R_1/2_) to near half-saturating flashes only at the highest dopamine concentration (250 μM). To better quantify effect magnitudes beyond *p*-values, we calculated percent changes, which express relative differences between values in control versus dopamine groups for parameters showing significant differences. We observed that exposure to 250 μM dopamine increased the R_1/2_ sensitivity of lamprey short photoreceptors by approximately 38% relative to control, whereas the increase in this parameter caused by the other two tested concentrations of dopamine did not exceed 6% (Figure 1E).

**Figure 2:**
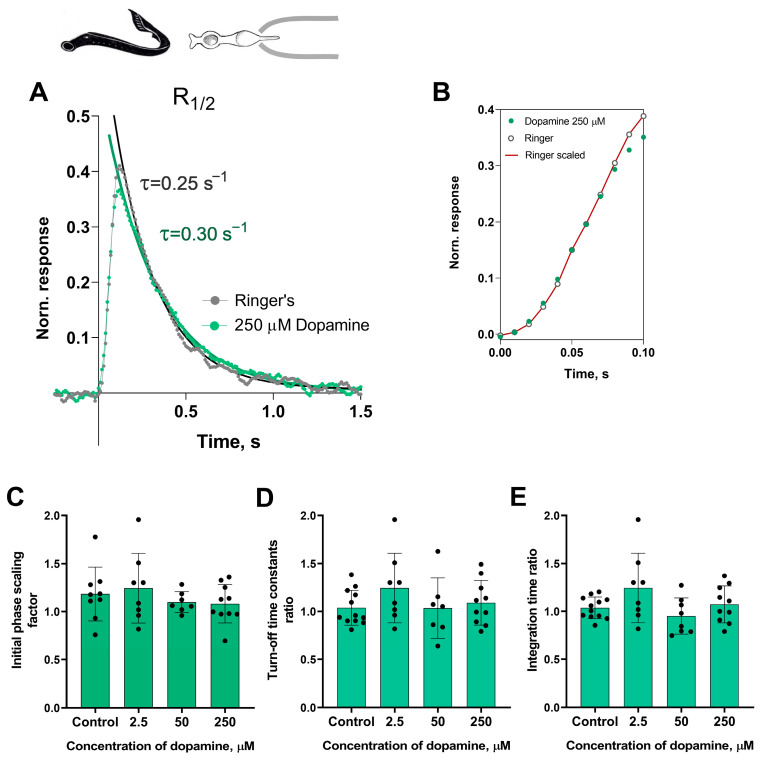
The effects of 2.5, 50, and 250 μM dopamine on the photoresponse kinetics of lamprey short photoreceptors. (**A**) An example of normalized R_1/2_ of lamprey short photoreceptors in Ringer’s solution (gray curve and dots), and after 30 min of 250 μM dopamine exposure (green curve and dots). (**B**) The result of scaling the rising phase of these two photoresponses: the red curve shows the result of multiplying the initial phase of the response in Ringer’s solution by a scaling coefficient of 1.0. Comparison of several response kinetic parameters: the scaling coefficient for the rising phase (**C**), response recovery rates (**D**), and integration time (**E**) for responses recorded in Ringer’s solution and after 30 min of dopamine exposure.

At first glance, it appeared that there were no significant changes in the initial or falling phases of the photoresponses of the short photoreceptors during dopamine exposure. Nevertheless, we decided to investigate this further. In order to analyze the behavior of the initial phase of the response, we only needed to determine the relative changes in the steepness of the initial phase under two experimental conditions. While we did not provide a mathematical description of the biochemical mechanisms behind activation, we extracted the ratio of two activation rate parameters by adjusting the rising phases of these two photoresponses, as shown in Figure 2B.

To estimate the rate of the reactions underlying the falling phase of the photoresponse, we fitted the falling phase of R_1/2_ with a single exponential function. We then extracted time constants τ, which were compared under two different conditions (see Figure 2A). We also compared the integration time of R_1/2_ in Ringer’s solution and following the application of dopamine. Figure 2C–E show a summary of the results from the 30 min dopamine exposure experiments on the kinetic parameters and integration time of short photoreceptor responses in comparison with controls. Regardless of the tested concentration, there were no effects of dopamine on the kinetic parameters after 30 min of incubation. It should be noted that, at the intermediate time point of 20 min, we observed no effects of any dopamine concentrations on the dark current, light sensitivities, activation and falling phases, or the integration time of non-saturated responses (data shown in Figure S1).

**Figure 3:**
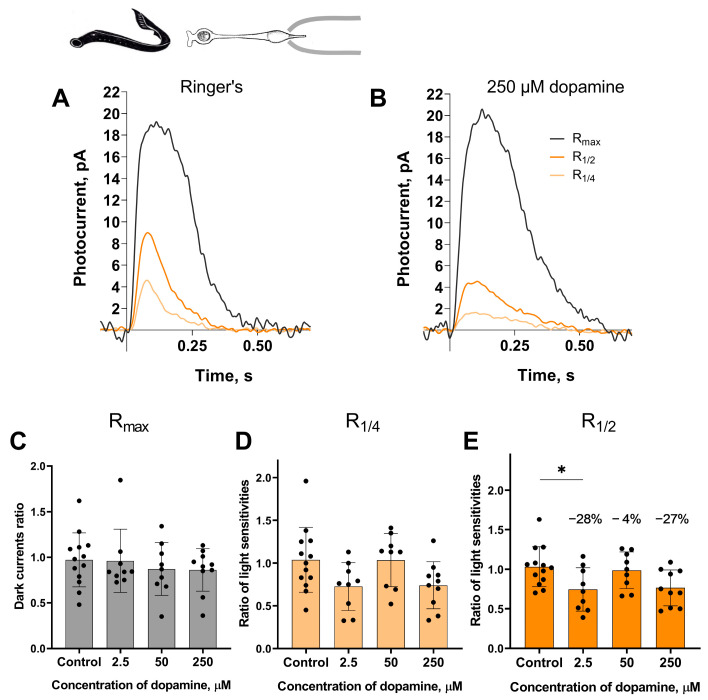
The effects of 2.5, 50, and 250 μM dopamine on lamprey long photoreceptors. A typical set of responses from lamprey long photoreceptors to saturating, near-half-saturating, and near-quarter-saturating flashes in Ringer’s solution (**A**), and after approximately 30 min of 250 μM dopamine exposure (**B**). Flash intensities are 3.27 × 10^4^ photons/μm^2^ per flash (saturating), 3.25 × 10^3^ photons/μm^2^ per flash (near half-saturating), and 1.29 × 10^3^ photons/μm^2^ per flash (near quarter-saturating); λ_max_ = 525 nm. Effects of dopamine at the tested concentrations on the dark current (**C**) and the light sensitivity to near quarter-saturating flashes (**D**) and half- saturating flashes (**E**), respectively. * *p* = 0.0436 (Dunnett’s multiple comparisons test).

*Long photoreceptors*. The experimental protocol for testing the putative effects of dopamine on the long photoreceptors in the river lampreys was, in general, similar to those described above for the short photoreceptors. A set of responses, including saturated, near half-saturated, and near quarter-saturated ones, were first recorded in Ringer’s solution. Then, recordings were made approximately 20 and 30 min after perfusion with Ringer’s solution containing 2.5, 50, or 250 μM dopamine. We analyzed the same parameters for the long photoreceptors as we did for the short ones. Figure 3 shows a typical set of responses from lamprey long photoreceptors: R_max_, R_1/4_, R_1/2_ in Ringer’s solution, and after 30 min of dopamine exposure.

At the 30 min time point, dopamine at a concentration of 2.5 μM reduced light sensitivity of R_1/2_ by approximately 28% relative to controls (Figure 3E). However, at the intermediate time point of the 20 min incubation (see Figure S2), no decrease in sensitivity was detected for any of the tested dopamine concentrations.

**Figure 4:**
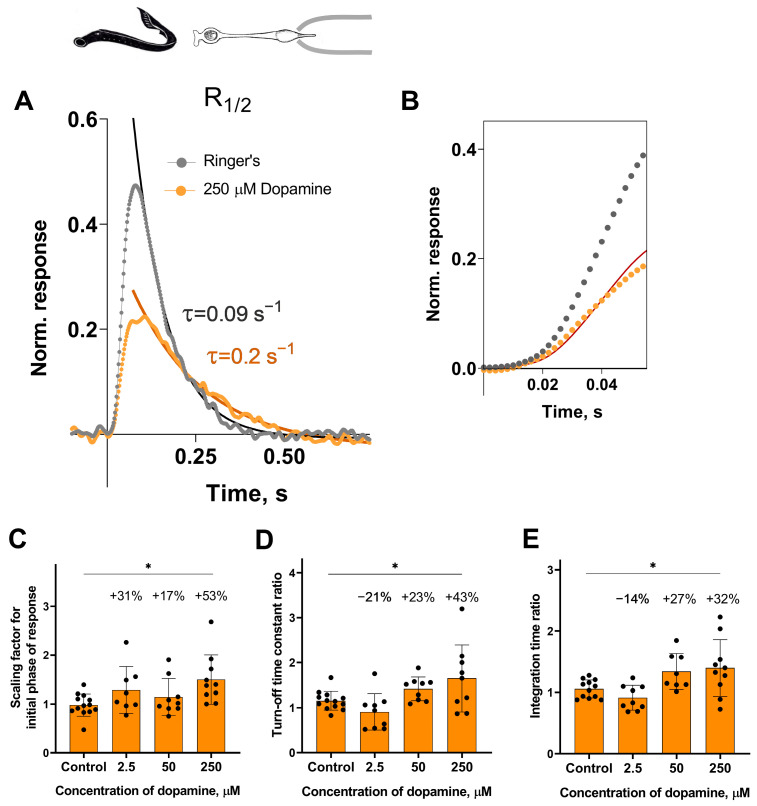
The effects of 2.5, 50, and 250 μM dopamine on the photoresponse kinetics of lamprey long photoreceptors. (**A**) An example of normalized R_1/2_ of lamprey long photoreceptors in Ringer’s solution (gray curve and dots) and after 30 min of dopamine exposure (orange curve and dots). (**B**) The result of scaling the rising phase of these two responses is shown by the red curve, which is the initial phase of the response in Ringer’s solution multiplied by a scaling coefficient of 0.54. Comparison of several response kinetic parameters: the scaling coefficient for the rising phase (**C**), the response recovery rates (**D**), and the integration time (**E**) for responses recorded in Ringer’s solution and after 30 min of dopamine exposure. * *p* = 0.0186, *p* = 0.0359, and *p* = 0.0389 for panels (**C**), (**D**), and (**E**), respectively (Dunnett’s multiple comparisons test).

Analysis of the response kinetics in the long photoreceptors revealed a statistically significant effect of the highest tested concentration of dopamine (250 μM) on all measured parameters after 30 min of incubation: it slowed down both the activation and turn-off rate constants (Figure 4C,D), and, as a result, increased the integration time of the non-saturated R_1/2_ photoresponses (Figure 4E). Compared to the control groups, the activation rate increased by ~53% (Figure 4C), the turn-off rate constant by ~43% (Figure 4D), and the integration time by ~32% (Figure 4E). At the 20 min time point, dopamine increased the turn-off rate constant by 38% and integration time by 45%, but did not affect the activation rate (see Figure S2D–F).

#### 2.1.3. The Lack of Effect of Increased cAMP Levels on the Photoresponse Properties in Both Long and Short Photoreceptors in the River Lamprey

We used the adenylcyclase activator forskolin to study the putative effects of increased intracellular cAMP levels on photoresponses of the river lamprey’s photoreceptors. Initially, photoresponses were recorded in Ringer’s solution. After that, the recordings were repeated after approximately 20 and 30 min perfusion with forskolin-containing solution (10 μM). The same light stimulation protocol was used for both sets of recordings. The tested sets of responses included (1) responses to a saturating flash—R_max_; (2) responses to a near quarter-saturating flash—R_1/4_; and (3) responses to a near half-saturating flash—R_1/2_. The fractional responses (R_1/4_ and R_1/2_) were normalized to the dark current measured at the corresponding time points, allowing parameter comparisons across experimental conditions. Normalized fractional responses were used to evaluate photoreceptor sensitivity from response amplitudes; they were also used to assess kinetic parameters. Dark current was quantified using the Rmax amplitude. We expressed all parameters as post-/pre-incubation ratios and compared them with values from control experiments.

**Figure 5:**
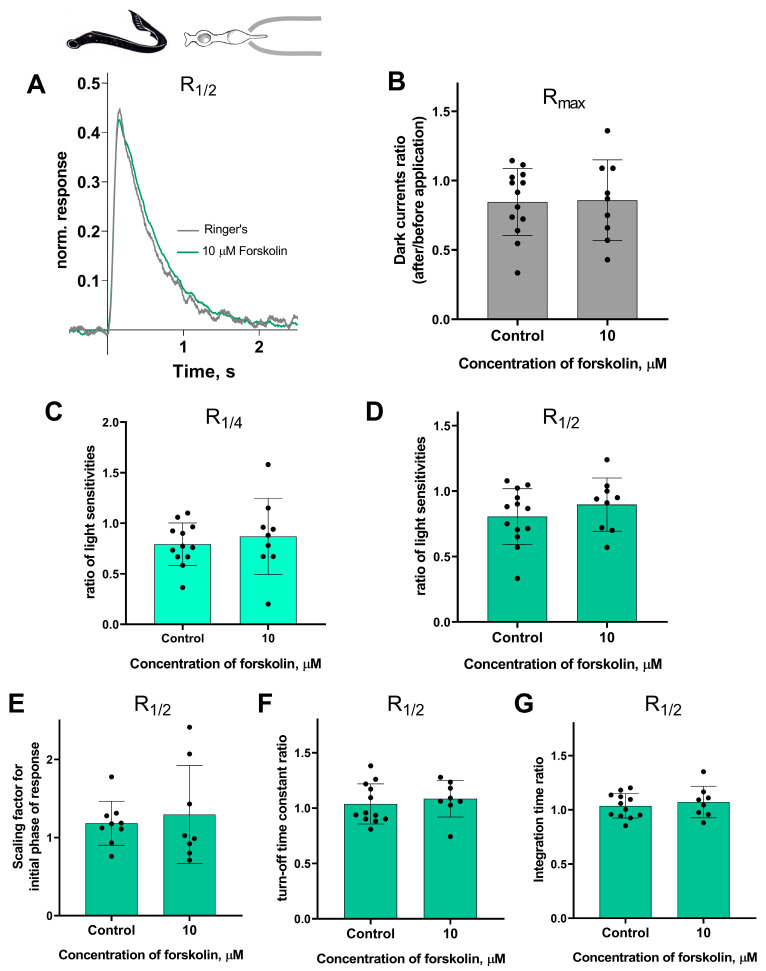
There is no effect of 10 μM forskolin on the photoresponses of short lamprey photoreceptors. (**A**) Responses of a representative short photoreceptor to near half-saturating flashes before (gray line) and after (green line) 30 min of forskolin application (each curve is an average of 8 records obtained under the same conditions). Flash intensity: 5.15 × 10^3^ photons/μm^2^ per flash, λ_max_ 525 nm. There is no difference in the dark current (**B**), light sensitivity of R_1/4_ (**C**), and R_1/2_ (**D**) for lamprey short photoreceptors after 30 min of forskolin exposure compared to the control experiments. There was no effect of forskolin on the kinetic parameters of R_1/2_: the scaling coefficient for the rising phase (**E**), the response recovery rates (**F**), and the integration time (**G**) after 30 min of forskolin exposure.

**Figure 6:**
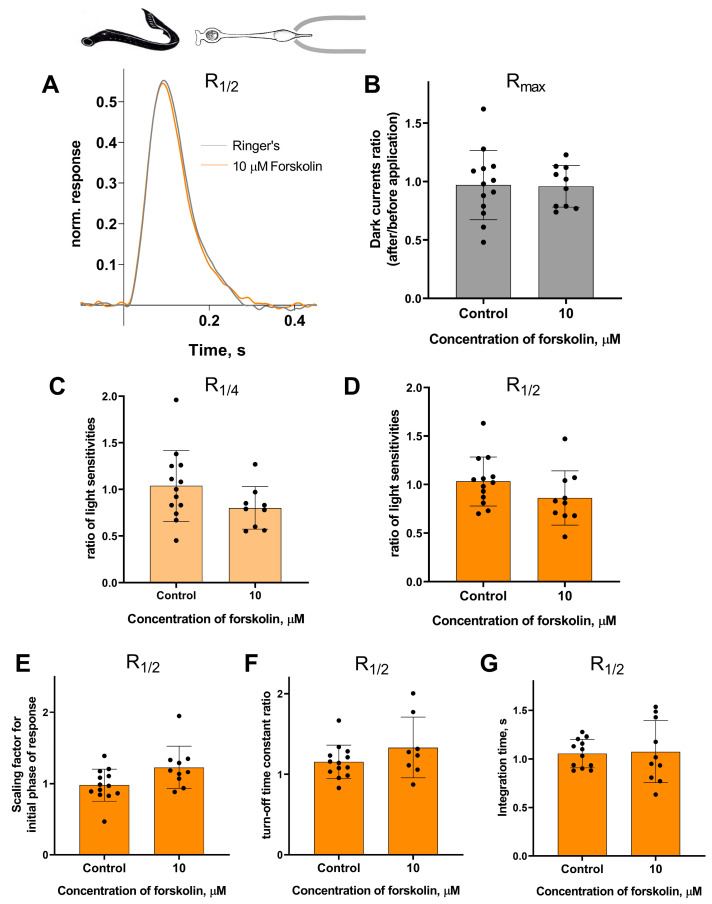
There is no effect of 10 μM forskolin on the photoresponses of long lamprey photoreceptors. (**A**) Responses of a representative long photoreceptor to near half-saturating flashes before (gray line) and after (orange line) 30 min of forskolin application (each curve is the average of 14 records obtained under the same conditions). Flash intensity: 5.15 × 10^3^ photons/μm^2^ per flash, λ_max_ 525 nm. There is no difference in dark current (**B**), light sensitivity of R_1/4_ (**C**), and of R_1/2_ (**D**) flashes for lamprey short photoreceptors after 30 min of forskolin exposure compared to control experiments. There was no effect of forskolin on the kinetic parameters of R_1/2_: the scaling coefficient for the rising phase (**E**), the response recovery rates (**F**), and the integration time (**G**) after 30 min of forskolin exposure.

Forskolin did not affect the sensitivity or the kinetic parameters of photoresponses to non-saturating flashes in both short and long lamprey photoreceptors. Figure 5 and Figure 6 show typical R_1/2_ before and after forskolin treatment. The response parameters in control and forskolin groups were very similar. This was true for both the final (30 min) and intermediate (20 min) time points (see Figures S3 and S4).

#### 2.1.4. The Lack of Dopamine Effect on the Photoresponse Properties in Fish Green-Sensitive Cones

In previous subsections, we described the clear effects of dopamine application on the long photoreceptors in the river lamprey. Here, we decided to compare the responses of lamprey long photoreceptors to dopamine with those of typical vertebrate cones, as these two types of photoreceptors are functionally similar. To this end, we selected isolated green-sensitive cones from the fish *Carassius gibelio*. This type of photoreceptor has been previously used in our long-term experiments and proved to be a stable and viable research subject [46].

In these experimental series, we tested the lowest and the highest concentration of dopamine (2.5 and 250 μM) on fish green-sensitive cones. The control experiments were conducted for green-sensitive fish cones using the same experimental protocol, but with Ringer’s solution only, in order to account for any potential changes in the main parameters of the photoresponses over time. We evaluated the same parameters as in the experiments involving lamprey long and short photoreceptors and took recordings at two time points: approximately 20 and 30 min after the start of the dopamine exposure.

**Figure 7:**
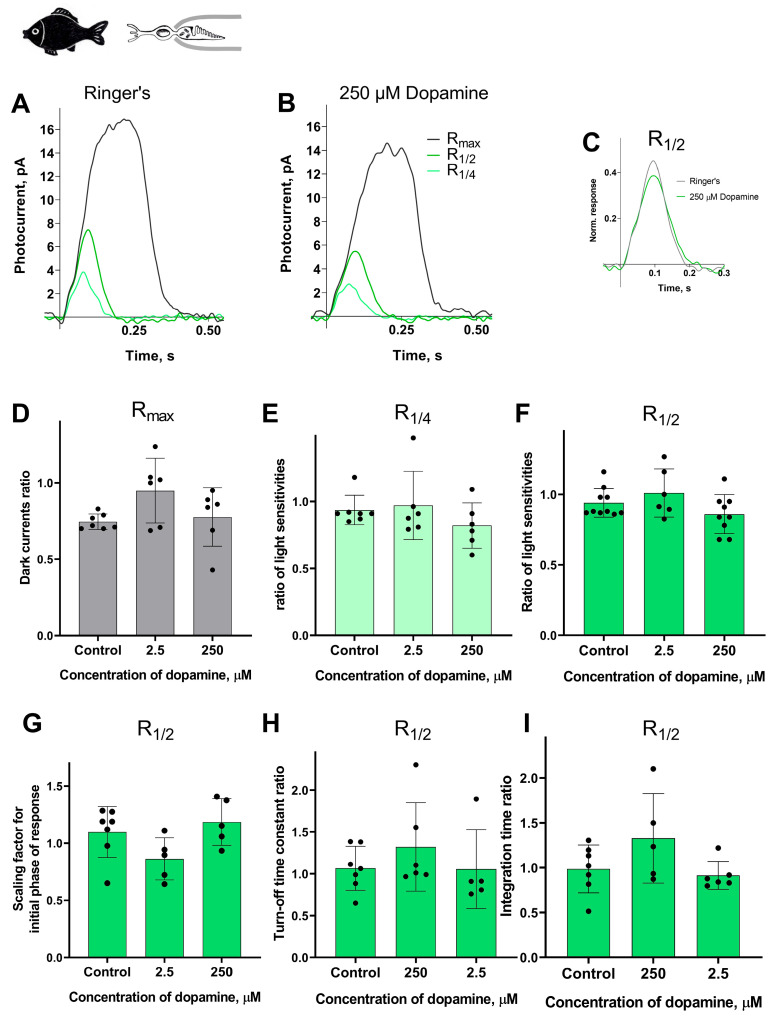
There is no effect of 2.5 and 250 μM dopamine on the green-sensitive cones of *Carassius gibelio*. A typical set of responses from a fish’s green-sensitive cone to saturating, near-quarter-saturating, and half-saturating flashes in Ringer’s solution (**A**) and after approximately 30 min of dopamine exposure (**B**). Flash intensities are 5.23 × 10^5^ photons/μm^2^ per flash (saturating), 1.23 × 10^4^ photons/μm^2^ per flash (near half-saturating), and 3.25 × 10^3^ photons/μm^2^ per flash (near quarter-saturating); λ_max_ = 525 nm. (**C**) An example of normalized R_1/2_ in Ringer’s solution (gray curve) and after 30 min of dopamine exposure (green curve), (**D**) and the light sensitivity of R_1/4_ (**E**) and R_1/2_ (**F**). Comparison of several response kinetic parameters: (**G**) scaling coefficient for the rising phase, (**H**) response recovery rates, and (**I**) integration time for R_1/2_ in Ringer’s solution and after 30 min of dopamine exposure.

Surprisingly, we observed no effect of dopamine on any of the measured parameters: dark current, light sensitivity, activation and turn-off kinetics, and integration time. A summary of the results of 30 min of dopamine exposure on fish green-sensitive cones is shown in Figure 7, and the results at the intermediate time point (20 min after the start of dopamine exposure) are shown in Figure S5. There were no significant differences in any of the tested parameters at either time point compared to the control experiments.

#### 2.1.5. Exposure of Dopamine to Outer Segments of Lamprey Long Photoreceptors and Carassius Cones

One possible explanation for the lack of dopamine effect on the light sensitivity of lamprey photoreceptors and green-sensitive cones of Carassius is that the drug was applied to the inner segments of these cells, but not to the outer segments, which in our typical experiment remained inside the pipette. In this study, we define the photoreceptor inner segment as the part of the cell surrounding the ellipsoid plus the myoid remaining after retinal dissection.

**Figure 8:**
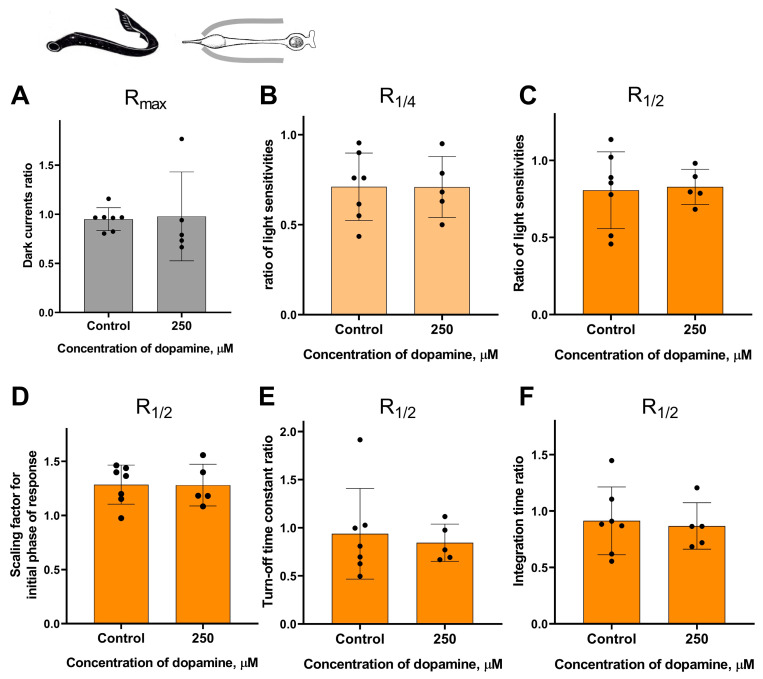
There is no effect of 250 μM dopamine on the photoresponses of long lamprey photoreceptors when applying to outer segments. There is no difference in the dark current (**A**), light sensitivity of R_1/4_ (**B**), and R_1/2_ (**C**) for lamprey short photoreceptors after 30 min of dopamine exposure to outer segments compared to the control experiments. There was no effect of dopamine on the kinetic parameters of R_1/2_: the scaling coefficient for the rising phase (**D**), the response recovery rates (**E**), and the integration time (**F**) after 30 min dopamine exposure to outer segments.

**Figure 9:**
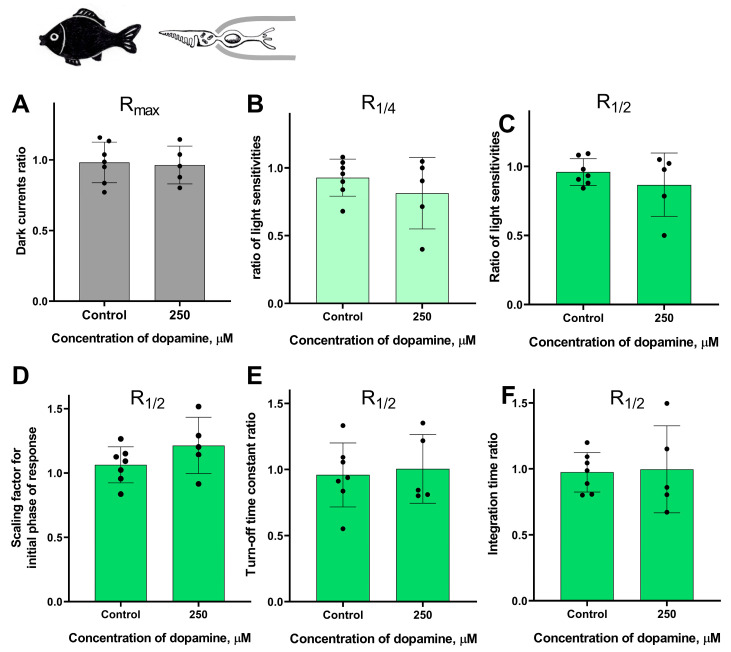
There is no effect of 250 μM dopamine on the photoresponses of Carassius green-sensitive cones when applying to outer segments. There is no difference in the dark current (**A**), light sensitivity of R_1/4_ (**B**), and R_1/2_ (**C**) for green-sensitive fish cones after 30 min of dopamine exposure to outer segments compared to the control experiments. There was no effect of dopamine on the kinetic parameters of R_1/2_: the scaling coefficient for the rising phase (**D**), the response recovery rates (**E**), and the integration time (**F**) after 30 min dopamine exposure to outer segments.

It should be noted that the survival rate of certain types of isolated photoreceptors in the experiment was much lower when the outer segment was exposed to the external washing solution compared to when it was kept inside the pipette. Nevertheless, despite the complexity of this task, we were able to study the potential effects of dopamine only at the highest concentration tested (250 μM) when applied to the outer segment for small samples of lamprey long photoreceptors and fish green-sensitive cones. These two series of experiments were performed in the same manner as for the “outer segment inward” configuration experiments, with time points of 20 and 30 min. For each series, a separate control group was included in the “outer segment outward” configuration for comparison. However, these two series of experiments did not reveal any statistically significant effect of 250 μM dopamine on the sensitivity, dark current, and kinetic parameters of photoresponses in either lamprey long photoreceptors (Figure 8 and Figure S6) or Carassius cones (Figure 9 and Figure S6).

Isolated short photoreceptors of lamprey, when sucked into a pipette in an “outer segment outward” configuration, demonstrated poor viability and did not survive the required protocol, which lasted at least 45 min per cell. Consequently, no data could be collected for lamprey short photoreceptors.

### 2.2. Immunoblotting and Immunohistochemistry

#### 2.2.1. Distribution of D1RD and D2RD in Photoreceptors and Other Retinal Structures in *Lampetra fluviatilis*

The retina of vertebrates contains the five conventional classes of neurons (photoreceptors, horizontal cells, bipolar cells, amacrine cells, and ganglion cells), which are organized into three main nuclear layers and two plexiform layers containing axons and dendrites. While the lamprey retina generally follows this organizational pattern, it has several specific morphological features [42]. In gnathostomes, ganglion cell axons converge at the vitreal edge of the retina to form the optic nerve, which extends into the central nervous system. In lampreys, however, the ganglion cells’ axons form an optic fiber layer (OFL) located between the inner nuclear layer (INL) and the inner plexiform layer (IPL) [47]. Most of the lamprey retina’s ganglion cells are displaced to the INL, with only a few observed within the IPL to form the inner ganglion cell layer (IGCL) [48,49]. Additionally, horizontal cells in the lamprey retina form two distinct sublayers (outer and inner horizontal cells, OHC and IHC), rather than being integrated into the INL as they are in gnathostomes [50]. Therefore, in the following text, we will use a different nomenclature for the layers of the lamprey retina compared to the retinas of fish and frogs.

Western blotting results demonstrate the presence of D1RD-immunopositive bands in the lamprey retina corresponding to 55 kDa and 100–130 kDa, which is essentially similar to the distribution of D1RD-immunopositive bands in the mouse retina (positive control, see Figure S7A). This suggests the existence of monomeric and dimeric forms of the receptor, as well as its various glycosylated states [51,52].

**Figure 10:**
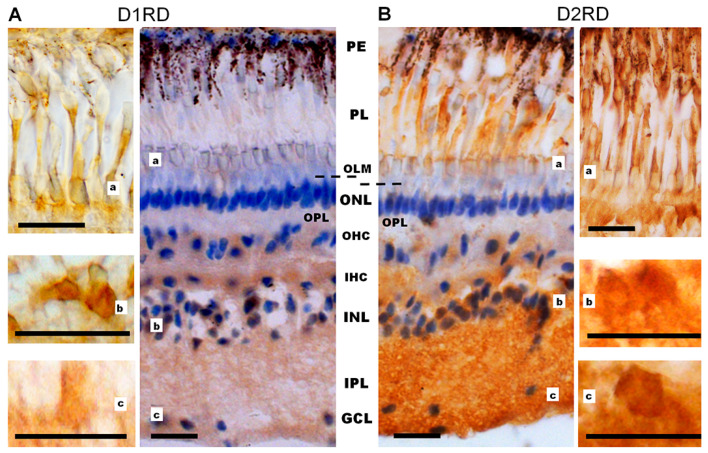
Cross-sections of the lamprey retina. Immunohistochemical reactions to D1RD (**A**) and D2RD (**B**). Counterstained with hematoxylin. Abbreviations (from [50]): PE—pigment epithelium; PL—photoreceptor layer; OLM—level of outer limiting membrane (*shown by dotted line*); ONL—outer nuclear layer; OPL—outer plexiform layer; OHC—outer horizontal cells; IHC—inner horizontal cells; INL—inner nuclear layer; IPL—inner plexiform layer; GCL—ganglion cell layer. a, b, c—representative images of D1RD (**A**) or D2RD (**B**) of the corresponding regions of the retina without hematoxylin staining. Scale bars—25 µm.

**Figure 11:**
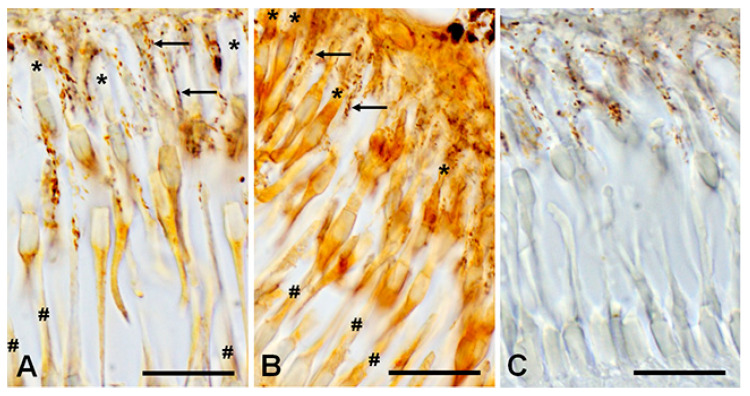
Immunohistochemical reaction to D1RD (**A**) and D2RD (**B**) in the photoreceptor layer (PL) of the lamprey retina and reaction without primary antibodies (**C**). Zoomed-in images obtained with an immersion objective 100×. Designations: *—outer segment of cones (long), #—outer segment of rods (shot), arrows—microvilli of pigment cells with pigment granules. Scale bars—20 µm.

Immunohistochemical analysis reveals a D1RD+ signal in various layers of the lamprey retina. A weak signal was detected in the photoreceptor layer (PL, includes only the outer and inner segments) in the outer segments of short, but not long, photoreceptors, and a brighter signal was detected in the myoid zone of the inner segments of both types of photoreceptors (Figure 10A, Figure 11A and Figure S10), while ellipsoid zones do not express D1. A weak reaction was also found in ONL in the perikaryons of photoreceptor neurons under OLM as well as in the OPL. A more intense reaction was noted in the cell bodies and neuropil of the INL: in the cell bodies of large OHC cells (no reaction was noted in the bodies of small cells) and in the cell bodies of the IHC. An intense reaction was noted in the cell bodies of large INL cells localized in the OGCL (Figure 10A). An intense reaction was also observed in the neuropil of the IPL layer and in the perikaryons and processes of ganglion cells located in the IGCL.

Western blotting results demonstrate the presence of immunopositive bands for D2RD in the lamprey retina that correspond to 100 kDa (as in the mouse retina), as well as additional bands above 55 kDa that are not detected in the mouse retina. This may indicate greater dimerization of D2RD in the mouse retina than in the lamprey retina, as well as the separate detection of S and L isoforms of D2RD in the lamprey retina (see Figure S7B).

Immunohistochemical analysis reveals an intense reaction to D2RD in various retinal layers (Figure 10B and Figure 11B). This reaction is present in the outer segments and myoid zone of inner segments of both short and long photoreceptors (PL), and a weak reaction is noted in the perikaryons of photoreceptor neurons (ONL) and in OPL. An intensive D2RD+ reaction is also observed in the perikaryons and neuropil of the INL and IPL layers. The most intense reaction is seen in the perikaryons of large cells (OGCL and IGCL) (see Figure 10B).

#### 2.2.2. Distribution of D1RD and D2RD in Photoreceptors and Other Retinal Structures in *Carassius gibelio*

Western blot results show a distribution of D1RD-immunopositive bands in the fish retina (50 kDa, 100 kDa, and 130 kDa regions) and additional bands (Figure S9A), similar to the distribution in the mouse retina.

**Figure 12:**
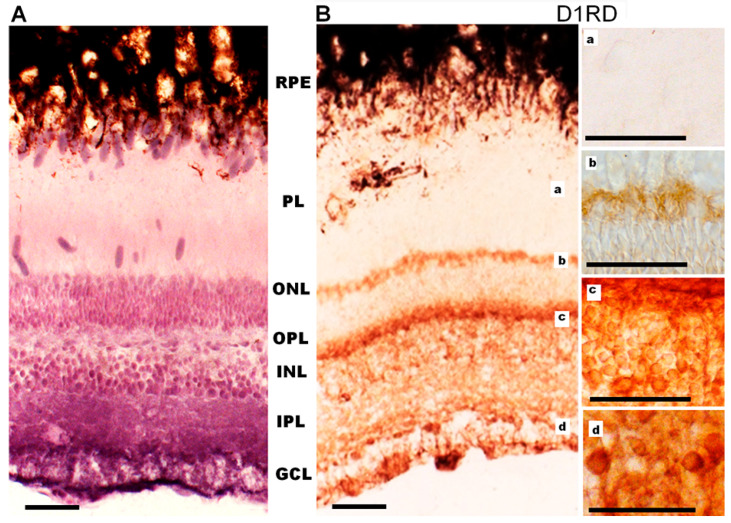
Cross-sections of the retina of *Carassius gibelio:* hematoxylin staining (**A**) and immunohistochemistry for D1RD (**B**). Abbreviations (from [54]): RPE—pigment epithelium; PL—photoreceptor layer; ONL—outer nuclear layer; OPL—outer plexiform layer; INL—inner nuclear layer; IPL—inner plexiform layer; GCL—ganglion cell layer. a, b, c, d—representative images of the D1RD from corresponding areas without hematoxylin staining. Scale bars—25 µm.

Immunohistochemical analysis of D1RD-immunopositive signal distribution in the Carassius retina shows no reaction in the main part of the PL. However, a distinct reaction is detected in the lower part of the PL at the border with the ONL (myoid part of the inner segment). In the ONL, the reaction is only detected in the perikaryons of cells located in the lower part of this layer (Figure 12). A weak reaction is detected in the OPL. In the INL, the reaction is detected in the perikaryons of cells, but the intensity varies between cells in this layer. A positive reaction to D1RD is evident in the IPL, particularly in the cells and processes of the GCL. The prevalence of D1RD-positive areas in the plexiform layers is consistent with previous immunohistochemical studies of fish retinas [34,53].

Western blotting analysis of the retina of *Carassius gibelio* revealed extremely weak D2RD− immunopositive bands at 100 kDa (Figure S9B) compared to the mouse retina, as well as more intense bands in the lower mass range (lower part of the gel).

**Figure 13:**
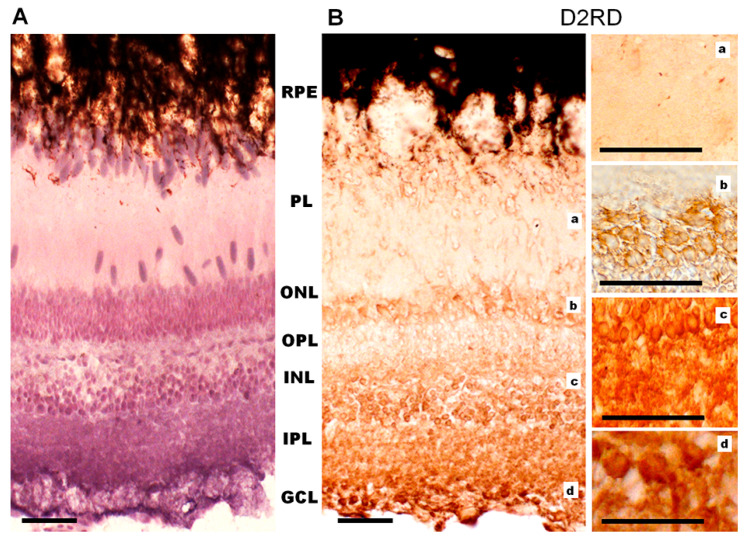
Cross-sections of the retina of *Carassius gibelio*: hematoxylin staining (**A**) and immunohistochemistry for D2RD (**B**). Abbreviations (from [54]): RPE: pigment epithelium; PL: photoreceptor layer; ONL: outer nuclear layer; OPL: outer plexiform layer; INL: inner nuclear layer; IPL: inner plexiform layer; GCL: ganglion cell layer. a, b, c, d—representative images of the D2RD from the corresponding areas. Scale bars—25 µm.

Immunohistochemical analysis of D2RD distribution in the Carassius retina (Figure 13) shows a weak D2RD+ reaction in the PL. In the ONL, a weak reaction is detected in the perikaryons of photoreceptors located in the lower part of this layer, and the most intense reaction is noted in the upper part of the ONL. A weak D2RD+ reaction is detected in the OPL, whereas a more intense and uniform reaction is noted in the perikaryons of INL cells. An intense D2RD+ reaction is also noted in the perikaryons and processes of GCL cells. A higher abundance of D2-like receptors than D1-like receptors has been observed previously [37], although the authors could not observe specific staining in the retina of Carassius species with the available antiserums.

#### 2.2.3. Distribution of D1DR and D2DR in Photoreceptors and Other Retinal Structures in *Pelophylax ridibundus*

Western blot results for the frog retina show a distribution of D1RD-immunopositive bands in the 50, 100, and 130 kDa regions, as well as the presence of additional bands similar to those in the mouse retina (Figure S9A). These data clearly indicate the presence of both monomeric and dimeric forms of the receptors, as well as their modifications.

**Figure 14:**
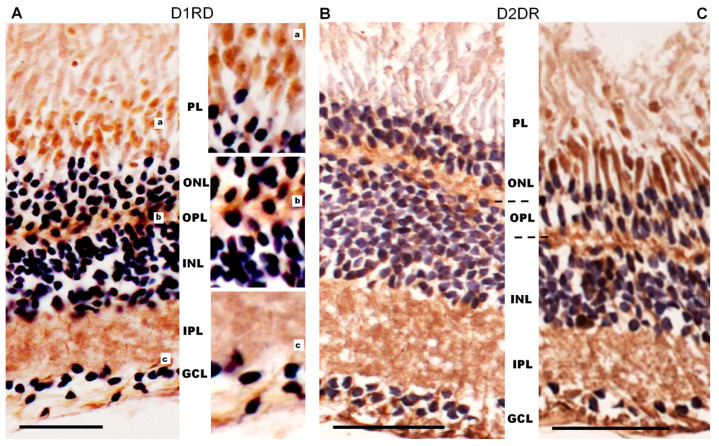
Cross-sections of the retina of the of a marsh frog (*Pelophylax ridibundus*) retina: immunohistochemistry for D1RD (**A**) and D2RD in the peripheral (**B**) and central (**C**) parts with hematoxylin staining. Abbreviations (from [54]): PL—photoreceptor layer; ONL—outer nuclear layer; OPL—outer plexiform layer (*shown by dotted line*); INL—inner nuclear layer; IPL—inner plexiform layer; GCL—ganglion cell layer. a, b, c—zoomed-in images of the corresponding areas. Scale bars—25 µm.

The immunohistochemical results for the frog retina (Figure 14A) show a D1RD+ reaction in the outer and inner segments of photoreceptors in the PL (mainly in the lower part, above the nuclei), as well as in the perikaryons of photoreceptors in the ONL. A D1RD+ reaction is clearly detected in the OPL and in some perikaryons located in the INL. An intense D1RD+ reaction is evident in the IPL, in the perikaryons, and in the processes of GCL cells. The detection of D1-like receptors in the retinal plexiform layers of frogs is consistent with earlier studies, while their potential localization to photoreceptors remains a matter of debate [53,55].

Western blotting analysis of the frog retina reveals D2RD-immunopositive bands around 100 kDa, which are similar to those in the mouse retina. This demonstrates that dimeric forms of D2RD are formed in both species (see Figure S9B).

Immunohistochemical analysis of the frog retina (Figure 14B) reveals a strong D2RD+ reaction in the inner segment sublayer of the PL, but this reaction is only detected in the central part of the retina. In the peripheral retina, as well as for the outer segments sublayer of the central, the PL only weakly reacts to D2RD. The D2RD+ reaction is clearly detected in the perikaryons of photoreceptors (ONL) and in the neuropil of the OPL, as well as in the perikaryons of individual neurons of the INL. An intense D2RD+ reaction is also detected in the IPL, as well as in the perikaryons and processes of ganglion neurons (GCL). This D2-like expression pattern matches earlier reports on the Xenopus retina [37].

## 3. Discussion

Our results suggest the existence of a dopamine-mediated regulatory mechanism that modulates the sensitivity and kinetics of lamprey photoreceptor responses to light. However, such a mechanism probably does not operate at physiological dopamine concentrations in the retina. This regulation differs markedly in nature from that observed in the rods and cones of jawed vertebrates. Notably, the intracellular cAMP-dependent regulation of the phototransduction cascade in lampreys is apparently absent or cannot be triggered by forskolin application.

### 3.1. Dopamine Modulation of Photoresponses in Jawed Vertebrates

The regulation of various aspects of gnathostome photoreceptor physiology by dopamine has been convincingly demonstrated (for reviews, see [11,56]). However, the role of dopamine in directly regulating the phototransduction cascade has only recently been evaluated [23], and its signaling pathways and the exact participants are still far from being fully resolved. It was reported that frog rod photoresponses remain unaffected by dopamine concentrations of up to 50 μM when applied to the inner segment. However, application to the outer segment produced significant modulation: sensitivity decreased, mainly due to a slower activation rate of the cascade, and, to a lesser extent, due to faster inactivation [23]. Fish cone photoresponses, as shown in the present study, were not sensitive to dopamine application to either the inner or outer segment, even at a high concentration of 250 μM.

Dopamine release in the retina changes cyclically, decreasing at night and increasing during the day or light exposure [57,58]. Based on the available data on the effect of dopamine on the phototransduction cascade, it can be assumed that its function is to adjust the sensitivity of the visual system to different light levels along with changes in illumination and the internal retinal circadian oscillator [59]. Indeed, dopamine receptors are found to be expressed in the retina of all major classes of jawed vertebrates [37,53]. Vertebrates possess several subtypes of dopamine receptors within the D1- and D2-like receptor families. In gnathostomes, the number of D1-like receptors varies from two (mammals) to four (teleost fish), and the number of D2-like receptors varies from three (mammals) to five (teleost fish) [60,61]. In this study, we analyzed the expression patterns of the two most common subtypes from each group: the D1 receptor (D1RD) and the D2 receptor (D2RD).

Photoreceptors are traditionally considered to predominantly express D2-like dopamine receptors and specifically D2RD [36,37,62], although some authors have also reported the presence of D1RD as well [34,63]. Our immunohistochemical analysis confirms the expression of both D1RD and D2RD in frog rods outer and inner segments. This is also consistent with the previous electrophysiological recordings following the application of selective dopamine receptor agonists: the decrease in sensitivity is most likely mediated by D1–D2RD heterodimers in the outer segment [23].

Importantly, the regulation of the phototransduction by dopamine appears to be specific to rods, while the photoresponses of cones remain unaffected. Our immunohistochemical analysis of dopamine receptor localization also reflects this, as we observed no expression of D1RD in the outer segments and ellipsoid areas of fish photoreceptors. The observed weak expression of the D2RD in the outer and inner segments is consistent with previous reports [34,37,53] and may reflect the involvement of dopamine regulation in retinomotor movements, as discussed in earlier studies [19,20]. The results of our electrophysiological experiments show that D2RD alone do not mediate any modulation of the phototransduction cascade.

### 3.2. Dopamine Modulation of Photoresponses in Lamprey

From an evolutionary perspective, lampreys are an incredibly interesting subject for studying retinal processes, as they are the most primitive vertebrates with a complex, multilayered retinal structure. The lamprey visual system also exhibits stepwise development throughout its complex life cycle, which is thought to reflect its evolutionary history [64]. In the larval stage, and during seasonal upstream migration in rivers, lampreys exhibit simple photophobic behavior and only actively move at night [65,66]). During most of the larval period their eyes are covered with skin, and only during metamorphosis do they transform into functional image-forming eyes [64,67].

Dopaminergic cells are also absent in the retina of early-stage larvae and only develop at a relatively late stage, close to metamorphosis [68,69]. This contrasts with jawed vertebrates such as fish and amphibians, whose amacrine cells actively express neurotransmitters already during the early larval period [70,71,72]. In adult lampreys, the organization of dopaminergic neurons follows a general pattern for vertebrates [73], with dopaminergic cell processes localized in the IPL (amacrine cells, see [68,74]), but not in both IPL and OPL (interplexiform cells). However, most vertebrates possess either a mix of both dopaminergic cell types or only interplexiform cells (e.g., teleost fish, some mammals; for a review, see [75]).

Although the photoreceptors of Northern Hemisphere lampreys can be functionally categorized as rods (short receptors) and cones (long receptors) [44,76], they retain several intermediate archaic morphological features [77,78] and biochemical properties of phototransduction cascade proteins [45,79]. The regulation of the phototransduction cascade by dopamine in lampreys differs significantly from that in gnathostomes as well. We have demonstrated that short photoreceptor responses remain unaffected by the application of 2.5 and 50 μM dopamine to the inner segment. However, a slight increase in sensitivity was observed when the concentration was increased to 250 μM, which is the opposite effect to that observed in gnathostome rods. Unlike fish cones, lamprey long photoreceptor responses were significantly modulated by the application of 250 μM dopamine to the inner segment. The on- and off-cascade processes slowed simultaneously, resulting in an increase in integration time. However, sensitivity to brief flashes of light remained unchanged. Remarkably, a significant decrease in sensitivity was observed after the application of a relatively low dopamine concentration of 2.5 μM, with no modulation of kinetics. Applying a high dopamine concentration to the outer segment of long receptors did not affect the photoresponses.

**Figure 15:**
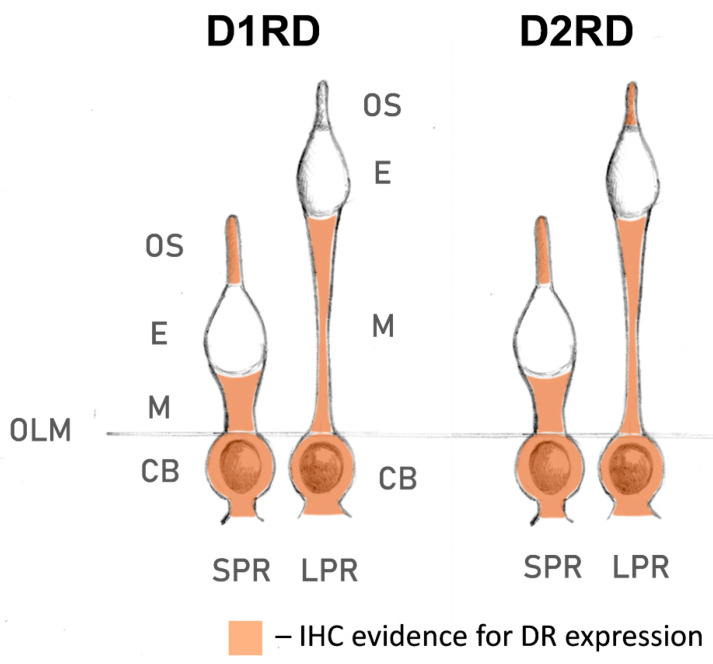
Localization of D1RD and D2RD dopamine receptors in river lamprey photoreceptors, according to immunohistochemical (IHC) analysis within the present study. Abbreviations: OLM—outer limiting membrane; SPR—short photoreceptor; LPR—long photoreceptor; CB—cell body; E—ellipsoid; M—myoid; OS—outer segment.

Lampreys have also been shown to possess fewer subtypes of dopamine receptors than gnathostomes: only one D1-like receptor and two D2-like receptors [80,81,82]. This may be due to the cyclostome branch separating before the second whole-genome duplication event that occurred in gnathostomes [83], resulting in their independent development since the Cambrian period. In this study, we have presented, to our knowledge, for the first time, data on the expression profile of dopamine receptors D1RD and D2RD in lamprey photoreceptors (summarized in Figure 15). Immunohistochemical analysis indicates that lamprey short photoreceptors show strong expression of D2RD, as well as weak expression of D1RD, in the outer segments. The outer segments of lamprey long photoreceptors contained only D2RD. In contrast to frog rods, neither photoreceptor type showed any dopamine receptor expression in the ellipsoid zone of inner segment, but a strong signal for both D1RD and D2RD was observed in the myoid. The role of these receptors is unclear as no retinomotor movements have been reported in the lamprey retina [84]. As the modulating effect of dopamine on the photoresponses was only observed when it was applied to the inner segment, the receptors located in the myoid are potential candidates that could mediate this effect. The presence of both D1RD and D2RD makes possible the formation of heterodimeric receptors, which allow dopamine modulation of phototransduction cascade in lamprey long receptors, in contrast to fish cones. Future research using subtype-specific dopamine receptor antagonists could help determine which exact receptors mediate the effect on photoresponses.

The slight increase in rod sensitivity and the decrease in the adaptive potential of cones under daylight conditions, which correspond to elevated retinal dopamine levels [1], may indicate that the visual system shifts into a high-sensitivity mode. This mechanism may be related to the specific activity cycle of lampreys during their seasonal upstream migration, when they burrow into the ground and minimize activity during the day, actively avoiding light [65]. It remains unclear whether a similar regulatory mechanism occurs during the active hunting phase in the sea.

As we observed most of the photoresponse modulation effects on lamprey photoreceptors only at extremely high dopamine concentration of 250 μM, one might question its physiological relevance for a living animal. Indeed, the average intercellular dopamine concentration in fish and amphibian retina was estimated to vary daily within only a 0.1–1 μM range [85,86]. Although the exact local retinal dopamine concentrations remain unknown, the lack of dopaminergic cell processes in lamprey OPL [74] suggests that high values are unlikely at the photoreceptor level [63]. Therefore, there is a probability that only the decrease in long receptor sensitivity at 2.5 μM dopamine represents a physiologically meaningful effect in the lamprey’s eye. This may reflect the fact that dopamine’s primary role in lamprey retina is the modulation of other aspects of photoreceptor circuitry and metabolism (gap junction coupling, glutamate and GABA receptors modulation, outer segment discs phagocytosis; see [11,56]) as opposed to phototransduction.

### 3.3. Role of cAMP in Dopamine Photoresponse Modulation: Lamprey vs. Gnathostomes

The key difference between the D1- and D2-like receptor families is that they have opposite effects on intracellular cAMP levels [28]. D1-like receptors increase adenylyl cyclase activity via the Gs pathway, whereas D2-like receptors decrease it via the inhibitory Gi pathway [87,88]. Additionally, dopamine can regulate intracellular cAMP via adrenergic receptors [89]. In protochordates, specifically ascidian larvae, modulation of photoresponses by dopamine occurs despite the absence of dopamine receptors [90,91]. As lampreys lack certain dopamine receptor subtypes [80,81], this adaptation may also apply to them. Cyclic day/night release of dopamine in the retina results in antiphase changes in the cAMP pool of photoreceptors [92,93]. The main target of intracellular cAMP level modulation is protein kinase A, which controls the phosphorylation of several key proteins in the phototransduction cascade [24,94]. Therefore, cAMP appears to be the most promising candidate for the role of key mediator in the regulation of photoresponses by dopamine.

Application of forskolin (2 μM) significantly increases frog rod sensitivity by slowing down the quenching processes of the phototransduction [24,95]. Interestingly, a similar effect on the photoresponses was observed in the cones of *Carassius gibelio* and zebrafish [25,27]; however, the increase in sensitivity was much less pronounced. An increase in intracellular cAMP concentration, which corresponds to the night-time period of the daily cycle, appears to provide an additional mechanism for adjusting rod sensitivity to low-light conditions. The decrease in the speed of cone adaptation mechanisms at night enhances their involvement in the visual process and may also cause them to switch to metabolic economy mode [25].

Notably, short and long lamprey photoreceptors are completely insensitive to forskolin application, even at higher concentrations (10 μM vs. 2 μM). At the same time, however, they showed changes in photoresponses after dopamine application, calling into question any involvement of intracellular cAMP levels in the modulation of the phototransduction cascade. In particular, the mechanism of slowing down cascade quenching associated with modulation of cone-specific opsin kinase GRK7 activity via PKA [26] can be ruled out for lamprey receptors. Dopamine-mediated modulation could be achieved through changes in calcium levels or direct interaction between dopamine receptors and certain ion channels [96,97]. Indeed, the decreased sensitivity observed in frog rods after dopamine application could not be fully explained by cAMP-mediated signaling alone and must also involve the regulation of intracellular calcium [23]. Dopamine receptors possess an additional signaling pathway based on the Gq protein, which triggers the massive release of calcium ions from intracellular stores [98,99]. This pathway may be responsible for the changes in lamprey photoresponses following the application of dopamine.

The absence of modulation of the phototransduction cascade at high levels of cAMP at nighttime may suggest distinct fundamental mechanisms of dopamine regulation in cyclostomes. Indeed, during their upstream migration to rivers, lampreys stop feeding [65], resulting in the gradual deterioration of the functionality of many signaling systems in their body cells [100]. To test this theory, we conducted experiments involving the application of forskolin to lampreys at various stages of the migration cycle, from initial entry into the river in November–December, through to spawning readiness in April–May, and beyond. Forskolin had no effect on lamprey receptor photoresponses at any stage, suggesting that insensitivity to elevated cAMP levels is a fundamental property of their phototransduction cascade.

It is important to note that our data can be alternatively interpreted as a lack of sensitivity of lamprey photoreceptor-specific isoform of adenylyl cyclase to forskolin. Thus, intracellular cAMP level in lamprey photoreceptors is not elevated, leaving only alternative signaling pathways for dopamine regulation of the phototransduction. Additionally, our results do not exclude the possibility of dopamine acting by reducing cAMP levels via D1-like receptors. The role of dopamine in regulating cyclostome photoreceptors responses clearly differs from its role in other vertebrates, and its mechanisms merit further research.

## 4. Materials and Methods

### 4.1. Experimental Animals and Dissection Procedures

Adult river lampreys (*Lampetra fluviatilis*) were caught in the rivers of the St. Petersburg vicinity in November 2020, 2021, and 2022, and were taken to the vivarium at the Sechenov Institute of Evolutionary Physiology and Biochemistry (IEPhB). They were kept in a cold room at 4–6 °C in large basins containing filtered, well-aerated water (25–30 L per animal) in the dark. Adult marsh frogs (*Pelophylax ridibundus*) were caught in the wild in southern Russia (the Astrakhan region) in September 2022 and 2023. They were then transported to the IEPhB vivarium, where they were kept under a layer of water in large tanks in refrigerators (at 4–6 °C) in the dark. The frogs were kept without food because their metabolic rate was greatly reduced in these conditions. Two days prior to the experiment, the frogs were removed from the refrigerator and placed on a natural day/night cycle. Adult Carassius (*Carassius gibelio*) were obtained from local hatcheries and kept in a 40 L aerated aquarium containing 5–7 fish at a time. The aquarium was kept in a laboratory room at 21–23 °C and maintained under a 12 h on/12 h off lighting cycle. The fish were fed spirulina-based dry fish food. Typically, animals were acclimatized for at least 48 h prior to scientific research.

For the electrophysiological studies and tissue extraction for Western blotting, all animals were dark-adapted overnight prior to each experiment. For histology, after overnight dark adaptation, the animals were exposed to moderate white illumination for 30 min to ensure tighter contact between the retina and the retinal pigment epithelium (RPE) and reduce detachment during dissection and fixed tissue processing. Additionally, it enabled the RPE microvilli to be unequivocally distinguished by the presence of melanin granules. They were then euthanized by decapitation and destruction of the spinal cord. The eyes were enucleated under dim red light. All subsequent dissection procedures (e.g., cutting the eyeball, removing the lens and cornea, and extracting the retina from the eyecup) were performed using a binocular magnifier with infrared illumination. The retinal samples were then used for either photocurrent recording experiments or immunohistostaining (see below for details of the methods).

The handling of experimental animals complied with the requirements of European Directive 2010/63/EU and the recommendations of the Bioethics Committee of the Sechenov Institute of Evolutionary Physiology and Biochemistry of the Russian Academy of Sciences (Permit #8/2021, issued on 26 August 2021).

### 4.2. Electrophysiology

#### 4.2.1. Preparation and Solutions

Electrophysiological experiments were conducted on the retinas of lampreys and fish. A piece of the isolated retina was transferred into a drop of Ringer’s solution (see composition below), shredded into small fragments with fine forceps, and gently pipetted several times to obtain isolated photoreceptors. As the lamprey vitreous humor is quite viscous, the retina was exposed to 0.25 mg/mL hyaluronidase for 2 min prior to shredding, to prevent clogging of the glass micropipettes. The resulting suspension of small retinal pieces and isolated photoreceptors was then transferred to a perfusion chamber in the experimental setup.

The retinal cells in the recording chamber were perfused constantly with the same Ringer’s solution that was used for dissection and storage of the retinal sample. The composition of the Ringer’s solution was species-specific. Lamprey: NaCl 120, KCl 3.6, MgSO_4_·7H_2_O 1.2, CaCl_2_ 1.1, NaHCO_3_ 22.6, HEPES 10, glucose 6; pH adjusted to 7.6. Frog: NaCl 90, KCl 2.5, MgCl_2_ 1.4, CaCl_2_ 1.05, NaHCO_3_ 5, HEPES 5, glucose 10, and EDTA 0.05, with the pH adjusted to 7.6. Fish’s Ringer’s solution contained the following in mM: NaCl 102, KCl 2.6, MgCl_2_ 1, CaCl_2_ 1, NaHCO_3_ 28, HEPES 5, and glucose 5, with the pH adjusted to 7.8.

The drug-containing solutions used in the experiments were 10 µM forskolin and 2.5, 50, and 250 µM dopamine, dissolved in Ringer’s solution. The forskolin solution was prepared from a fresh stock solution (10 mM) in dimethyl sulfoxide (DMSO) on the day of the experiment. The final concentration of DMSO in the perfusion solution was less than 0.1%, which had no negative effect on photoreceptor viability or electrical activity. The dopamine solution was made up from powder immediately prior to application to the tested photoreceptor, as dissolved dopamine is susceptible to oxidation over time. All chemicals were purchased from Sigma-Aldrich (St. Louis, MO, USA).

The preparation procedures and subsequent electrophysiological recordings were carried out at a room temperature of 17–19 °C.

#### 4.2.2. Single-Cell Recordings and Experimental Protocol

The responses of single photoreceptors were studied using the suction micropipette technique [101] with some modifications. Details of our suction rig and its operation can be found in our previous publications [46,102].

Glass suction micropipettes were pulled using a micropipette puller (P-97 Flaming/Brown Micropipette Puller, Sutter Instrument, Novato, CA, USA). In a perfusion chamber of the setup, a photoreceptor was pulled into the micropipette. Most experiments were conducted in the configuration where the photoreceptor outer segments faced inward the micropipette. For some experiments, we managed to record from lamprey long receptors and Carassius green-sensitive cones in the configuration where the outer segments faced outward. We usually recorded from single photoreceptors (short and long) protruding from the edge of the lamprey retina, or from isolated cones (green-sensitive members of double cones) in the case of the fish retina.

The cells were stimulated with 10 ms (rods) or 2 ms (cones) of LED light at λ_max_ = 525 nm. Second-channel stimulation with red (λ_max_ = 630 nm), green (λ_max_ = 525 nm), or blue (λ_max_ = 460 nm) lights enabled unambiguous identification of green-sensitive fish cones, distinguishing them from red- and blue-sensitive types. Cone spectral sensitivity was determined by analyzing the response-intensity curves obtained from photoreceptor responses to flashes ranging from minimal to saturating intensities at each wavelength. The emission spectra of all the light stimuli used in the setup were recorded using a USB4000 spectrometer (Ocean Optics, Orlando, FL, USA). LED intensities were measured at the same positions where the preparations were located using an OPT-301 optosensor (Burr-Brown Corporation, Tucson, AZ, USA). Flash intensity was regulated using switchable neutral density filters inserted into the light path. The diameter of the light stimulus spot at the level of the isolated photoreceptor suspension, where stimulation and response recordings were conducted, was 1 mm. In this zone, the stimulus intensity is uniform and each photoreceptor from all the animal species studied falls within the boundaries of this spot by a large margin. Photoresponses were recorded at 100 Hz for rods and 500 Hz for cones, and were low-pass filtered at 30 Hz using an eight-pole analogue Bessel filter. Data acquisition, stimulus timing, and flash intensity were controlled by LabVIEW hardware and software (National Instruments, Austin, TX, USA).

The light stimulation protocol was identical for all photoreceptor types and recording configurations; it included stimulation by light flashes that elicited maximum (saturated) and fractional responses (approximately one-quarter and one-half saturated). For each cell, light intensities that produced the desired fractional responses were selected individually based on the maximum response amplitude. The light intensities of the responses varied depending on the species, photoreceptor type, and recording configuration. Specifically, in the lamprey short photoreceptors, light intensities eliciting saturated responses (R_max_) varied from ~0.2 × 10^4^ to ~2.6 × 10^4^ photons/μm^2^ per flash, those eliciting 1/4 responses (R_1/4_) varied from ~14 to ~310 photons/μm^2^ per flash, and those eliciting 1/2 responses (R_1/2_)—from ~62 to ~647 photons/μm^2^ per flash. In the lamprey long photoreceptors (recording configuration “outer segment in”), the light intensities were as follows: for R_max_ ~3.27 × 10^4^–26.1 × 10^4^ photons/μm^2^ per flash, for R_1/4_ ~0.21 × 10^3^–4.09 × 10^3^ photons/μm^2^ per flash, and for R_1/2_ ~0.41 × 10^3^–8.17·× 10^3^ photons/μm^2^ per flash. The ranges of light intensities recorded from the lamprey long photoreceptors, in ″outer segment out″ configuration, were ~3.27 × 10^4^–13.1 × 10^4^ photons/μm^2^ per flash (R_max_), ~0.21 × 10^3^–2.1 × 10^3^ photons/μm^2^ per flash (R_1/4_), and ~0.41 × 10^3^–4.1 × 10^3^ photons/μm^2^ per flash (R_1/4_). In green-sensitive cones of Carassius, the following light intensities were used: (recording configuration “outer segment in”) for R_max_ ~0.33 × 10^5^–5.23 × 10^5^ photons/μm^2^ per flash, for R_1/4_ ~65–3.25 × 10^3^ photons/μm^2^ per flash, and for R_1/2_ ~325–1.23 × 10^4^ photons/μm^2^ per flash; (recording configuration “outer segment out”) R_max_ ~0/33 × 10^5^–1.31 × 10^5^ photons/μm^2^ per flash, for R_1/4_ ~62–1.63 × 10^3^ photons/μm^2^ per flash, and for R_1/2_ ~103–4094 photons/μm^2^ per flash. The experimental protocol included several steps. Firstly, R_max_, R_1/4,_ and R_1/2_ were recorded sequentially in Ringer’s solution. Then, the perfusion solution was replaced with a solution containing forskolin or dopamine (the time required to completely change the solution in the perfusion chamber is approximately 2.5 to 3 min). The responses were re-recorded according to the light stimulation protocol after 20 and 30 min of incubation in drug-containing solution. The same protocol was applied to control experiments (no drug added) to quantify how cellular metabolic rundown affected response amplitude over time. Thus, for each cell, two sets of responses were recorded: one in Ringer’s solution and one in drug-containing solution (or in Ringer’s solution again for controls), and all parameters are presented as ratios of post-incubation to pre-incubation values.

#### 4.2.3. Data Processing and Statistical Analysis

Data captured in the setup were processed using custom software written in LabVIEW. To estimate putative changes in photoreceptor responses, we analyzed their dark current, sensitivity, kinetics of the rising and falling phases, and integration time. The dark current was measured as the absolute peak response (R_max_) produced by a saturating flash. The sensitivity was assessed by the magnitude of the normalized (to the dark current) responses to subsaturating stimuli that elicited responses at approximately 25% and 50% of the saturated response (R_1/4_ and R_1/2_, respectively). The activation rate is defined by the steepness of the response rising phase. In this study, we focused mostly on quantifying the relative changes in the steepness of the rising phase, without assuming mathematical models of the underlying biochemical mechanisms. Therefore, for each cell we calculated the ratio of two activation rate parameters by choosing a scaling factor that maximized the convergence of the curves for the normalized (to the dark current) fractional responses in their rising phases. The falling phase of the fractional responses, which reflects the main deactivation processes in the phototransduction cascade, was fitted using a single exponential function to obtain a turn-off time constant. The rising and falling phases of photoresponses were analyzed using custom-made Python software (version 3.8). To determine the integration time, fractional responses were first normalized to their own peak amplitudes (scaled to 1) and then integrated over time. The ratio values of parameters in the dopamine and forskolin groups were compared to those in the control group using appropriate statistical tests, because these represented independent samples. Statistical analyses were carried out using GraphPad Prism (version 10.6.1). The normality of datasets from lamprey and fish photoreceptor light responses was confirmed using the Shapiro–Wilk test. Parameters in the control and drug-containing solution groups were compared using either a one-way ANOVA with a post hoc Dunnett’s multiple comparisons test or an unpaired *t*-test. A *p*-value of less than 0.05 was taken to indicate statistical significance. The multiple comparisons correction took into account both dopamine and forskolin application groups, as they were compared to the same control group. Data in all figures are presented as individual ratio values, with bars indicating the mean ± standard deviation.

The sample size (n) indicates the number of individual photoreceptors tested per experimental group, including controls. The values of n varied from 5 to 13 cells, consistent with standard practice in our research area. From each retina of a single eye, we prepared 2–3 specimens for suction-pipette recordings. This allowed us to record from up to six cells per animal in some experiments. Cells that did not survive the entire protocol were excluded from further analysis. In total, data were collected from at least three animals per group, typically four to five. For immunohistochemistry and immunoblotting, the retinas of four animals of each species were used. Control and dopamine/forskolin experiments were conducted alternately, with animals randomly assigned to groups to ensure unbiased distribution.

### 4.3. SDS-Polyacrylamide Gel Electrophoresis (SDS-PAGE), Immunoblotting

The dissected retinas (4–5 from each species) were homogenized in the ratio 1:10 in the lysis buffer containing the following: 20 mM Tris-HCl (pH 7.5), 150 mM NaCl, 2 mM EDTA, 2 mM EGTA, 0.25 M sucrose, 0.5% Triton X-100, 0.5% sodium deoxycholate, 15 mM NaF, 10 mM sodium glycerophosphate, 10 mM sodium pyrophosphate, 1 mM Na3VO4, 1 mM phenylmethylsulfonyl fluoride (PMSF), 0.02% NaN3, and the protease inhibitor cocktail (Sigma-Aldrich, USA). The homogenates were then centrifuged for 15 min at 11,000× *g* and 4 °C. The resulting supernatant was collected, and the proteins in the sample were solubilized by boiling in Laemmli sample buffer. The SDS-PAGE and immunoblotting protocols have been described previously [103]. Briefly, the protein samples were fractionated by SDS-PAGE in 10% and 12% polyacrylamide gels, then transferred to 0.22 μm nitrocellulose membranes (GE Healthcare, Amersham Biosciences AB, Little Chalfont, UK) by electroblotting (300 mA for 1 h) in a mini trans-blot module (“Bio-Rad Laboratories Inc.”, Hercules, CA, USA). Non-specific binding was blocked by incubating the membranes in 5% non-fat dry milk diluted in TBST (0.1% Tween-20 in Tris-buffered saline) for 1 h at room temperature. The membranes were washed with TBST and then incubated overnight at 4 °C with primary antibodies diluted in blocking solution: rabbit anti-D1 dopamine receptor (D1RD; Abcam, Cambridge, UK; 1:1000) or rabbit anti-D2(L/S) dopamine receptor (D2RD; Millipore, Darmstadt, Germany; 1:500). After removing the primary antibodies, the membranes were incubated with horseradish peroxidase (HRP)-conjugated goat anti-rabbit IgG (Sigma; 1:10,000 dilution) for 1 h at room temperature. The membrane was washed with TBST three times for 10 min each time. The Novex ECL Chemiluminescent Substrate Reagent Kit (Invitrogen, Carlsbad, CA, USA) and premium X-ray film (GE Healthcare, Little Chalfont, UK) were used to visualize the bands.

### 4.4. Preparation of the Retina Sections

Immediately after dissection, the eyecups (4–5 from each species) were immersed in 4% paraformaldehyde solution (Sigma, Burlington, MA, USA) at 4 °C for 30 min, then washed with 0.9% sodium phosphate buffer (PBS) and immersed in 10% and 30% sucrose solution in PBS at 4 °C overnight. The retinas were then frozen on dry ice using Tissue-Tek medium (Sakura Finetek Europe, Alphen aan den Rijn, The Netherlands) and stored at −80 °C. Cross-sections 10–20 µm thick were prepared using a Leica CM1510 cryostat (Leica Microsystems, Wetzlar, Germany). A series of slices were made from different zones of each retina, and every fifth slice was mounted on a SuperFrost/Plus glass (Menzel, Rüthen, Germany) (20–30 slices). The glasses were left to dry overnight at room temperature before being used for immunohistochemistry or hematoxylin staining.

### 4.5. Immunohistochemistry

The slides with retina sections were washed with PBS, treated with a 0.6% hydrogen peroxide solution in PBS for 30 min to block endogenous peroxidase activity, then washed with PBS for 15 min. Finally, they were washed with a PBS solution containing 0.1% Triton X-100 (PBST) for a further 30 min. The sections were then incubated for 1 h in a blocking solution (a mixture of 3% goat serum and 2% bovine serum in PBST). The sections were incubated with either the primary rabbit anti-D1 dopamine receptor antibody (Abcam; dilution 1:300) or the primary rabbit anti-D2 receptor antibody (Millipore; dilution 1:200) in a 2% blocking solution for 48 h at 4 °C. After washing in PBST for 40 min, the sections were incubated for one hour in PBST with goat anti-rabbit biotin-conjugated IgG (Vector Laboratories, Inc., Newark, CA, USA) at a dilution of 1:600. After washing in PBS, the sections were incubated for 1 h in a streptavidin-peroxidase solution at a dilution of 1:1000 (Sigma, USA) in PBS. After washing in PBS, the sections were treated with a solution of 0.05% diaminobenzidine (Sigma-Aldrich, St. Louis, MO, USA) and 0.03% hydrogen peroxide in PBS. The reaction was stopped using distilled water. After washing, the sections were mounted in glycerol under coverslips. Some sections were stained with hematoxylin. The specificity of the immunohistochemical reaction was verified using a negative control (samples without primary antibodies; see Figure S8).

### 4.6. Microscopy

Micrographs of retina sections were obtained using a Carl Zeiss Imager A1 microscope with transmitted light (Axio Vision 4.7.2 software, Carl Zeiss, Jena, Germany) and an Axiocam 712 video camera (using Zen 3.4 Blue Edition software, Carl Zeiss, Oberkochen, Germany). The images were obtained using objectives EC Plan-NeoFluar 20×/0.5, 40×/0.75 and 100×/1.3 oil at room temperature. PowerPoint and Photoshop CS6 were used to prepare the demonstration material. During image processing, operations such as rotation, brightness/contrast, cropping, and zooming were used.

## Data Availability

The original contributions presented in this study are included in the article/Supplementary Materials. Further inquiries can be directed to the corresponding author.

## Supplementary Materials

The following supporting information can be downloaded at: https://www.mdpi.com/article/10.3390/ijms27031435/s1.

## Abbreviations

The following abbreviations are used in this manuscript:

cAMP	Cyclic adenosine monophosphate
D1RD	D1 receptor
D2RD	D2 receptor
PE	Pigment epithelium
PL	Photoreceptor layer
OLM	Level of outer limiting membrane
ONL	Outer nuclear layer
OPL	Outer plexiform layer
OHC	Outer horizontal cells
IHC	Inner horizontal cells
INL	Inner nuclear layer
IPL	Inner plexiform layer
GCL	Ganglion cell layer
