# Supplementary material for: Differences in the Effect of Dopamine on the Phototransduction Between Lampreys and Jawed Vertebrates
Source: Int J Mol Sci. 2026 Jan 31;27(3):1435. doi: 10.3390/ijms27031435 (PMC12897906; doi:10.3390/ijms27031435)
Supplement: Supplementary file 1 [file ijms-27-01435-s001.zip › ijms-4039475-supplementary.pdf]

Supplementary materials to MS

Differences in the effect of dopamine on the phototransduction between lampreys and jawed vertebrates

Darya A. Nikolaeva, Alexander Yu. Rotov, Irina Yu. Morina, Michael L Firsov, Irina V. Romanova and Luba A. Astakhova

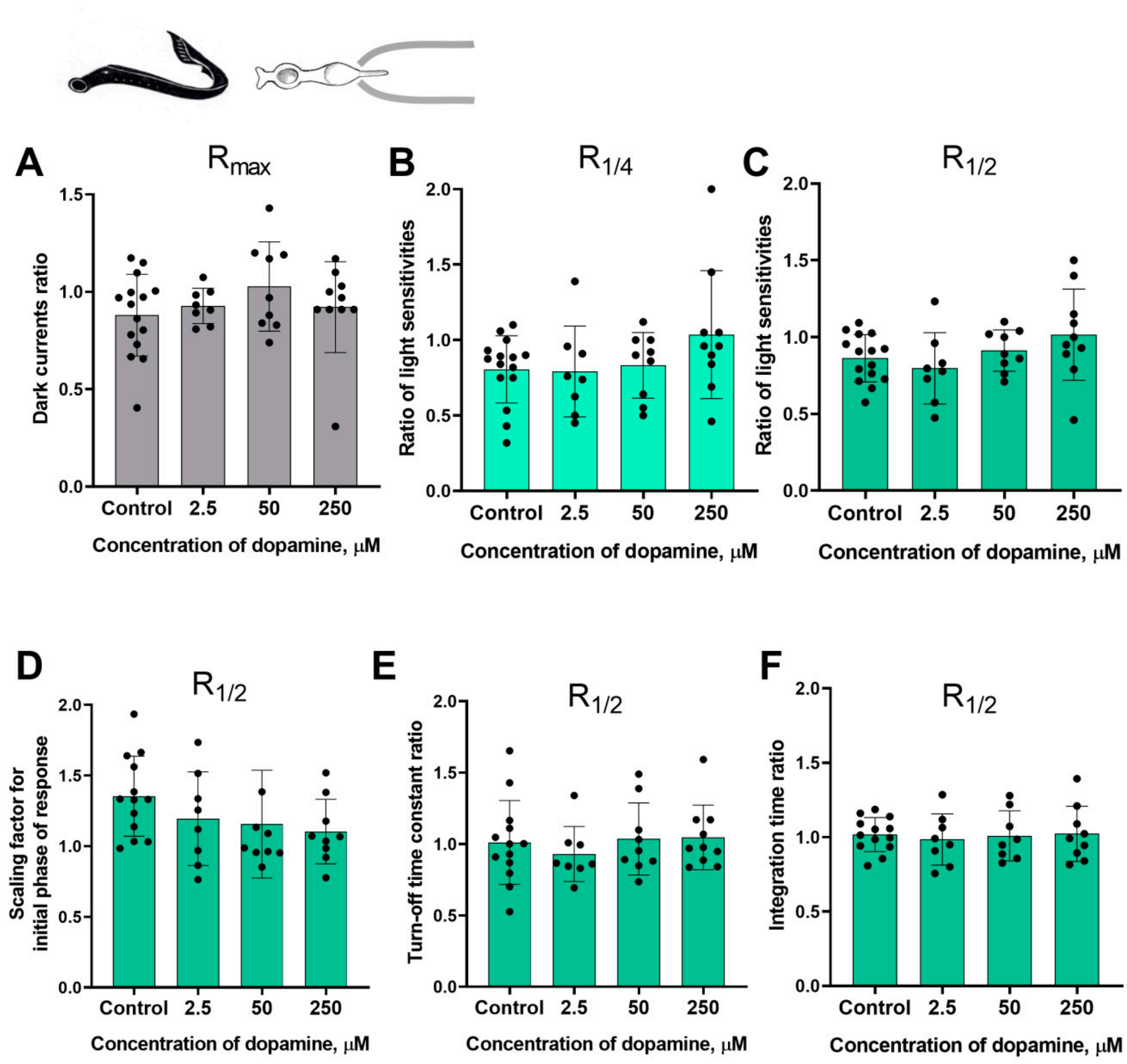

Figure S1. Effects of 2,5, 50 and 250 μM dopamine on the dark current, light sensitivity and photoresponse kinetics of lamprey *short* photoreceptors **after approximately 20 minutes' exposure (first time point in dopamine)**. Comparison of the dark current (A) light sensitivity to quarter-saturating (B) and half-saturating (C) flashes, the scaling coefficient for the rising phase (D), the response recovery time

constant (E) and the integration time (F) for half-saturated responses recorded in normal Ringer's solution and after a 20-minute exposure to dopamine. No statistically significant differences were observed using one-way ANOVA and the post hoc Dunnett test.

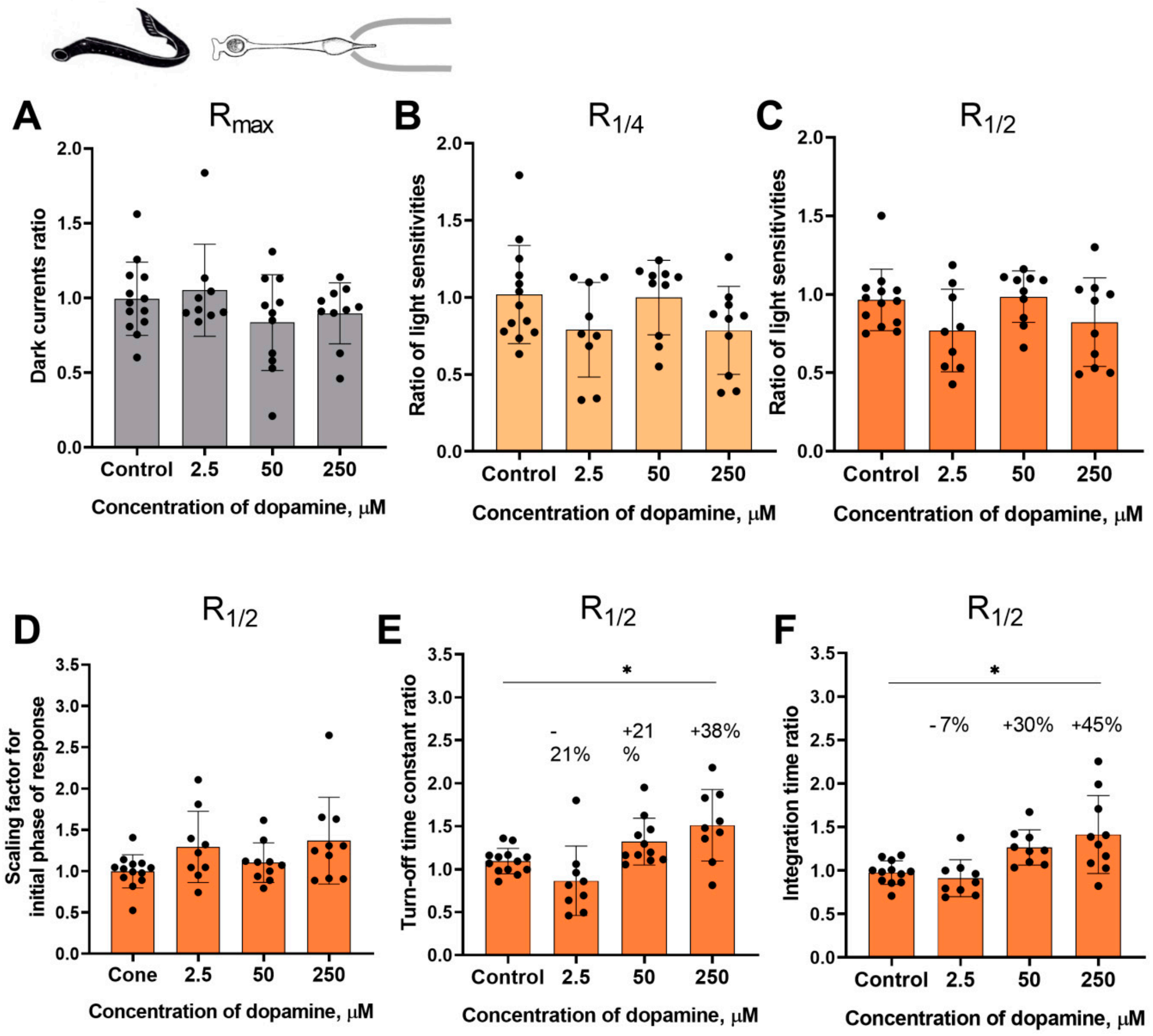

Figure S2. Effects of 2,5, 50 and 250 μM dopamine on the dark current, light sensitivity and photoresponse kinetics of lamprey *long photoreceptors* **after approximately 20 minutes' exposure (first time point in dopamine)**. Comparisons of dark current (A) and light sensitivity to near quarter-saturating (B) and half-saturating flashes (C), for responses recorded in normal Ringer's solution and after a 20-minute exposure to dopamine, showed no statistically significant differences (one-way ANOVA and post hoc Dunnett's test). Scaling coefficient for the rising phase (D), response recovery rates (E), and integration time (F) of half-saturated responses significantly increased for cells incubated in a solution containing 250 μM dopamine compared to the control group ($p = 0.098$ and $p = 0.038$, for panels E and F, respectively. Dunnett's multiple comparisons test).

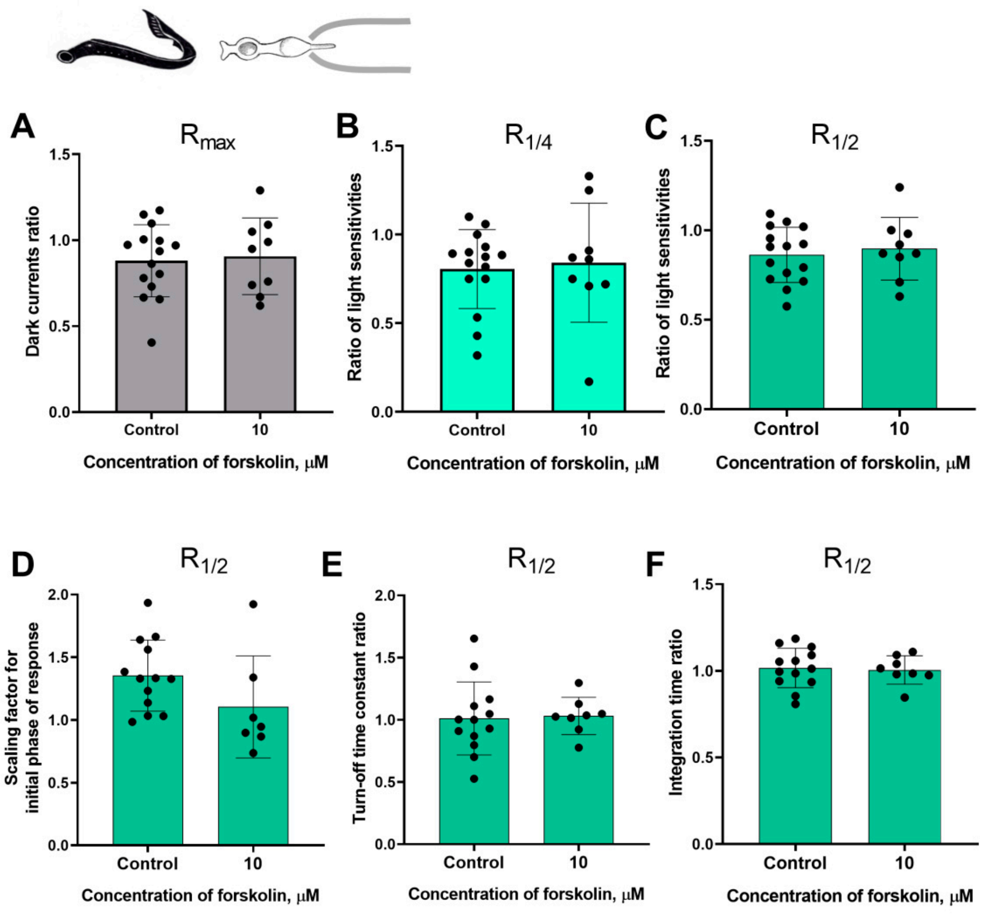

Figure S3. The effects of 10 μM forskolin on the dark current, light sensitivity and photoresponse kinetics of lamprey *short photoreceptors* **after approximately 20 minutes' exposure (first time point in forskolin)**. The graph shows a comparison of the dark current (A), light sensitivity to near quarter-saturating (B) and half-saturating (C) flashes, the scaling coefficient for the rising phase (D), response recovery rates (E) and integration time (F) for half-saturated responses recorded in normal Ringer's solution and after 20 minutes of forskolin exposure. No statistically significant differences were observed using an unpaired t-test.

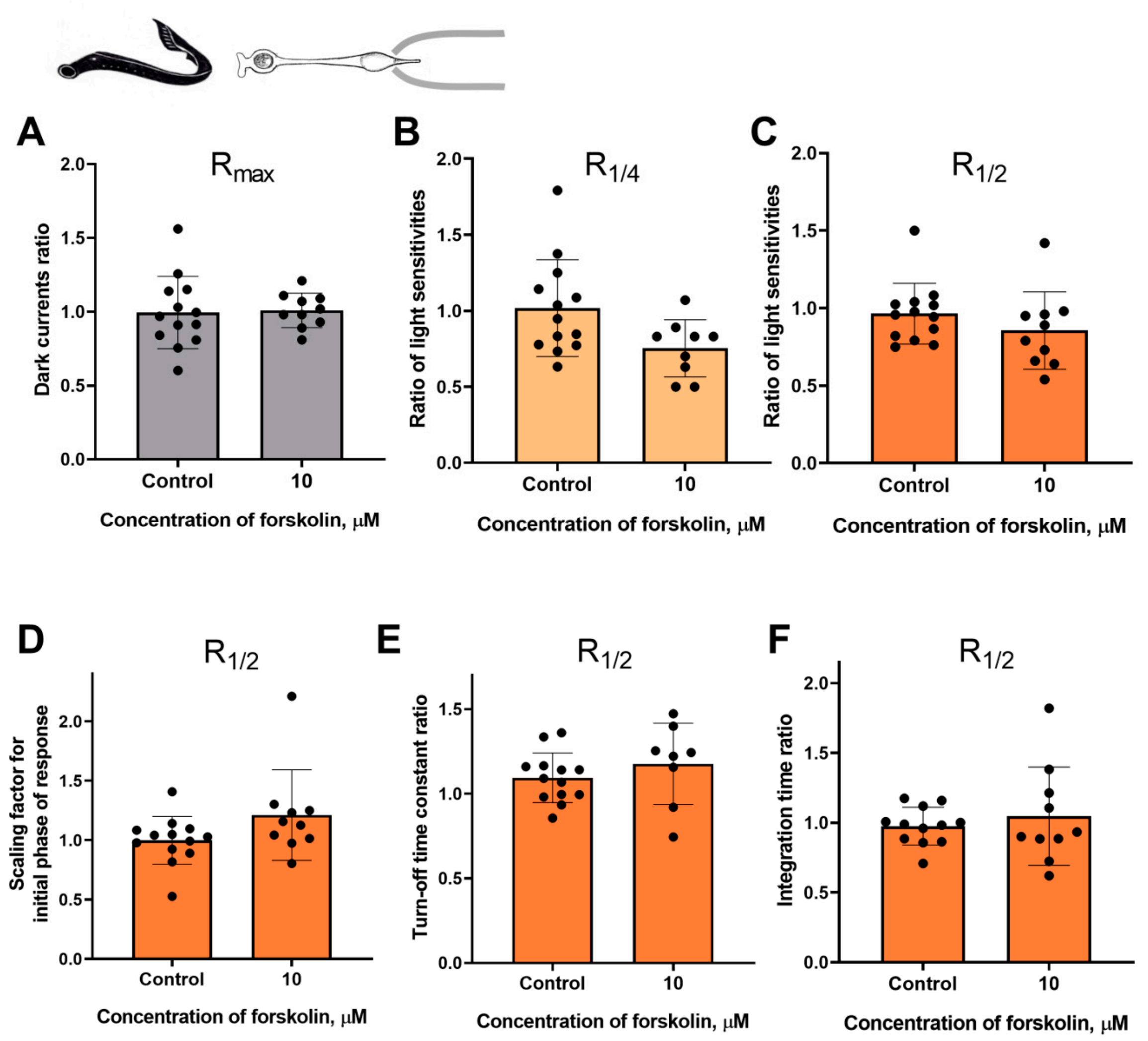

Figure S4. The effects of 10 µM forskolin on the dark current, light sensitivity and photoresponse kinetics of lamprey <u>*long photoreceptors*</u> **after approximately 20 minutes' exposure (first time point in forskolin)**. This figure shows a comparison of the dark current (A), light sensitivity to near quarter-saturating (B) and half-saturating flashes (C), the scaling coefficient for the rising phase (D), response recovery rates (E) and integration time (F) for half-saturated responses recorded in normal Ringer's solution and after 20 minutes of forskolin exposure. No statistically significant differences were observed using an unpaired t-test.

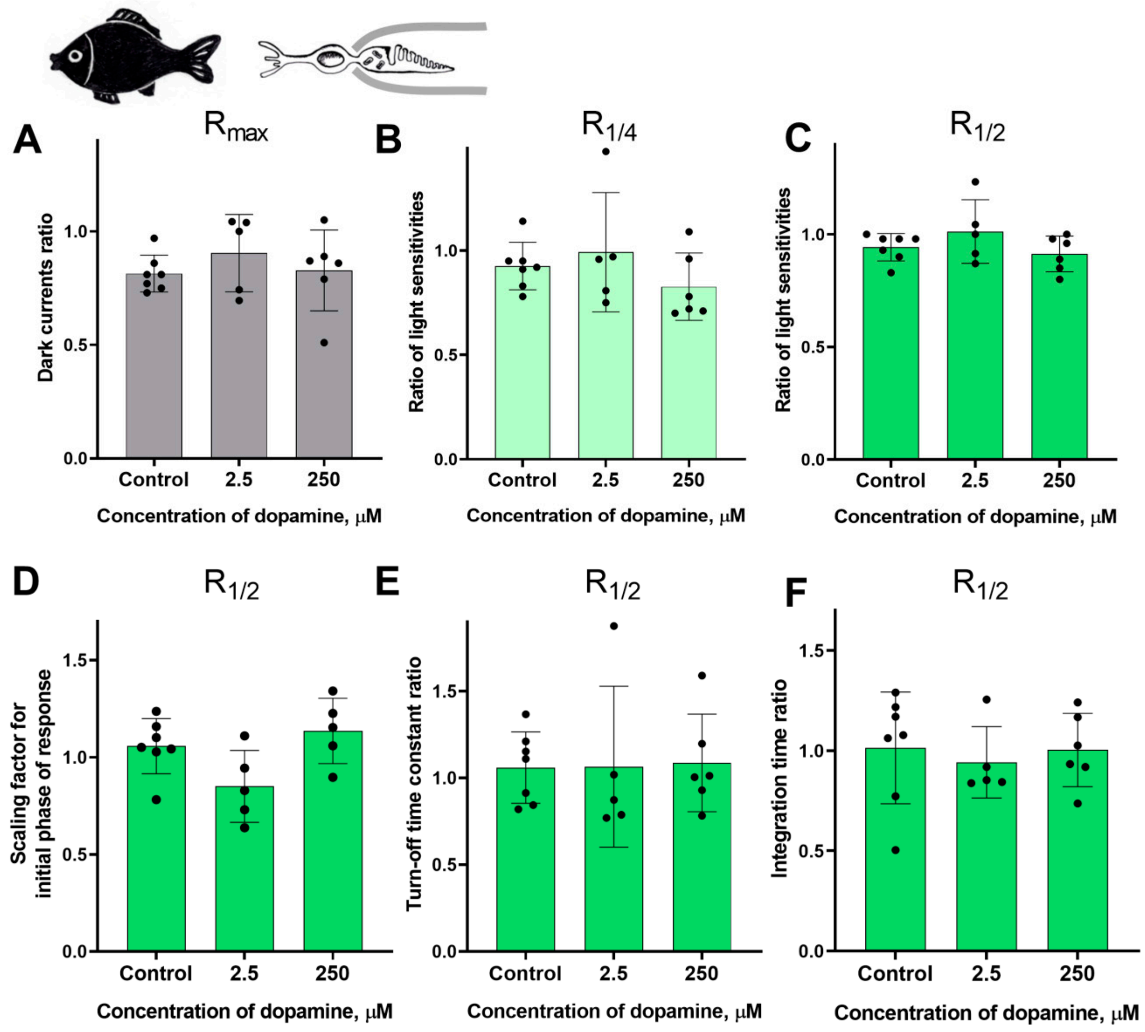

Figure S5. Absence of effect of 2.5 and 250 μM dopamine on green-sensitive cones of the fish *Carassius gibelio*: no changes observed after 20 minutes of dopamine exposure in dark current (A) and light sensitivity to near quarter-saturating (B) and half-saturating flashes (C). A comparison of several response kinetic parameters: a scaling coefficient for the rising phase (G), the response recovery rates (H), and the integration time (I) for half-saturated responses recorded in normal Ringer's solution and after 20 minutes of dopamine exposure. No statistically significant differences were observed using an unpaired t-test.

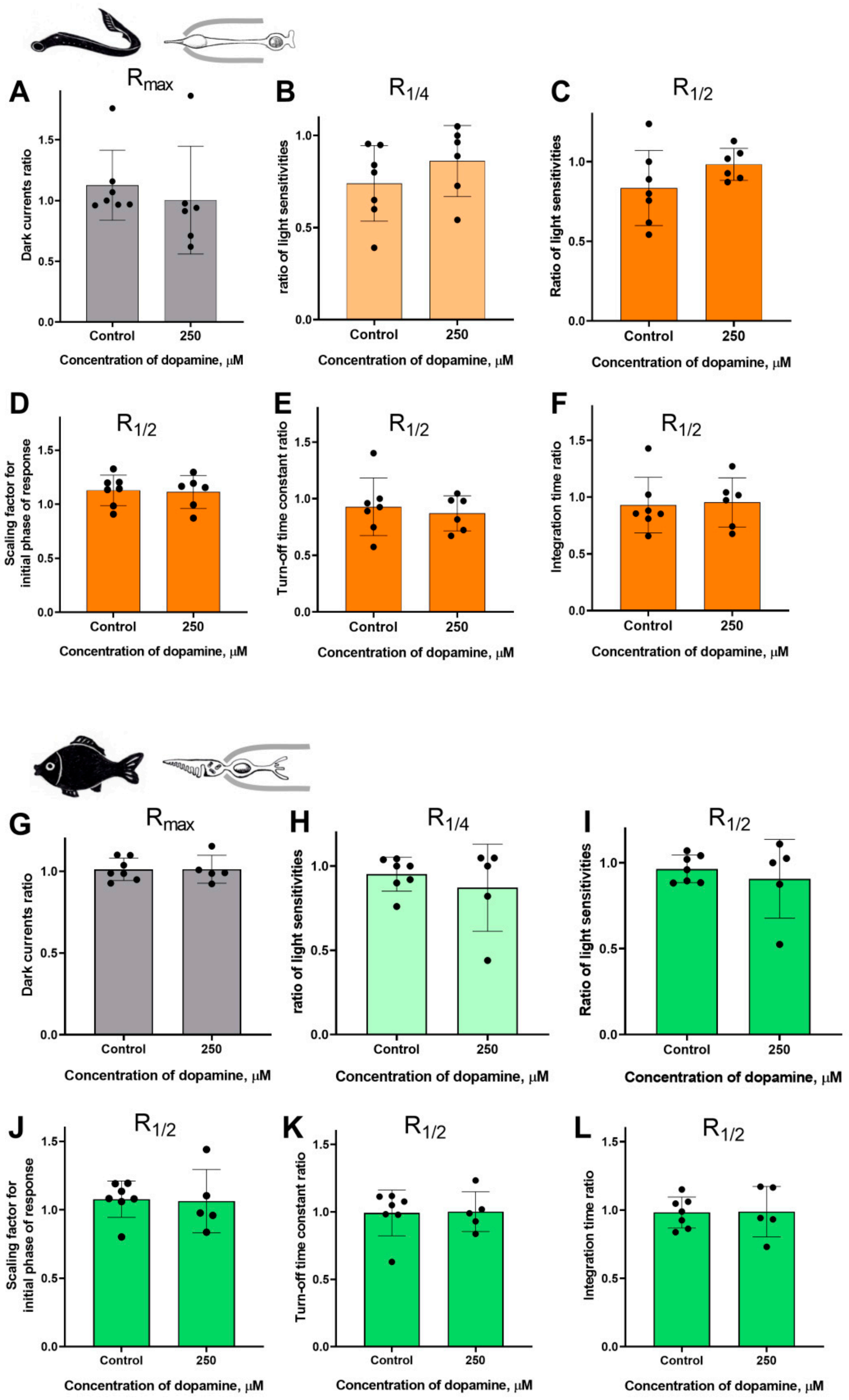

Figure S6. Absence of effect of 250 µM dopamine on the photoresponses of long lamprey photoreceptors (**A-F**) and green-sensitive cones of the fish (**G-L**) when applying to outer segments. There is no difference in the dark current (**A, D**), light sensitivity to near quarter-saturating flashes (B, H), or half-saturating flashes (**C, I**) for both types of photoreceptors after 20 minutes of dopamine exposure to outer segment compared to the control experiments. There was no effect of dopamine on the kinetic parameters of the half-saturated response: the scaling coefficient for the rising phase (**D, J**), the response recovery rates (**E, K**) and the integration time (**F, L**) after 20 minutes dopamine exposure to outer segments.

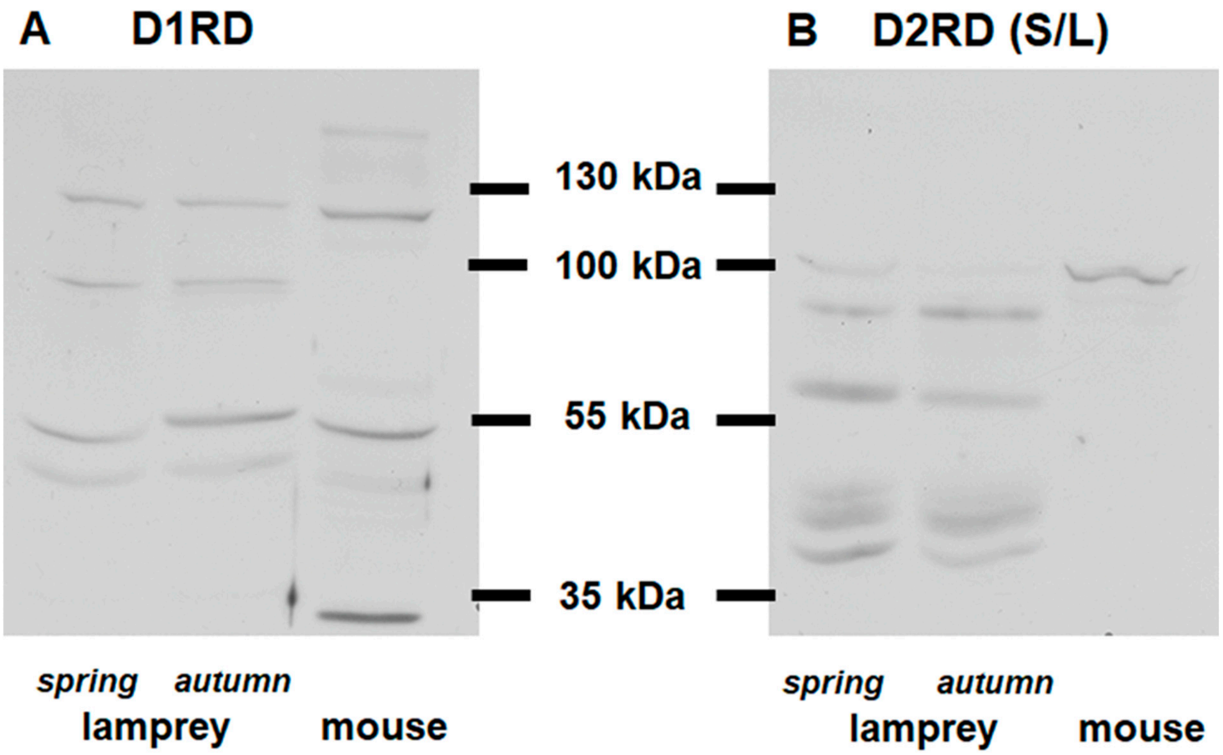

Figure S7. Western blotting results demonstrate the presence in the lamprey retina (A) D1RD-immunopositive bands in regions around 55 kDa and between 100 and 130 kDa and (B) D2RD-immunopositive bands in the 100 kDa region and additional bands in the 55 kDa region. The mouse retina was used as a positive control. Lamprey retinas for Western blotting were obtained in both spring and autumn to detect potential seasonal variations; however, no differences were observed between these two time points.

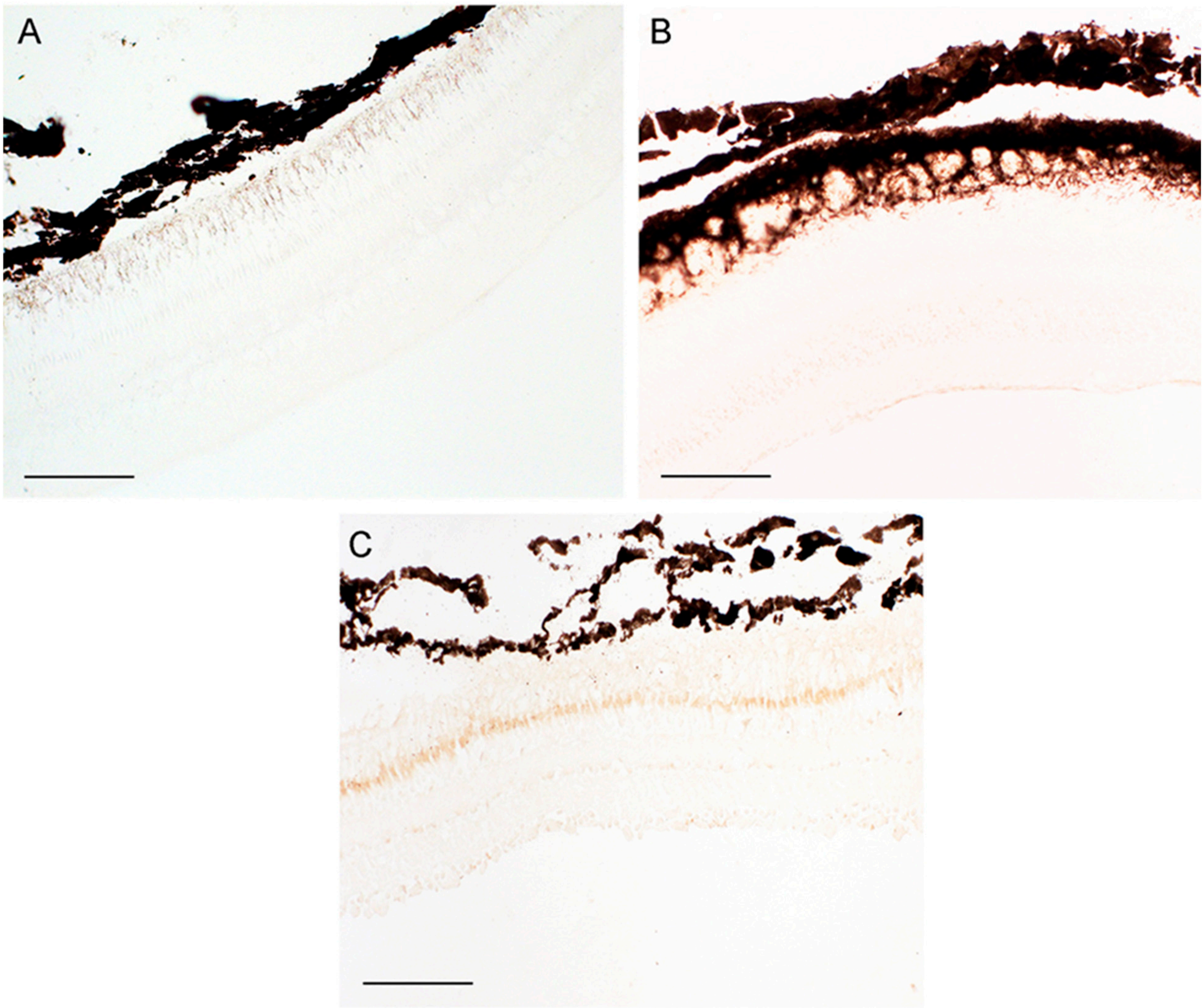

Figure S8. Negative controls for immunohistochemical reactions (i.e. reactions without primary antibodies) in the retinas of lamprey (A), carassius (B) and frog (C). Scale bars - 100 μm.

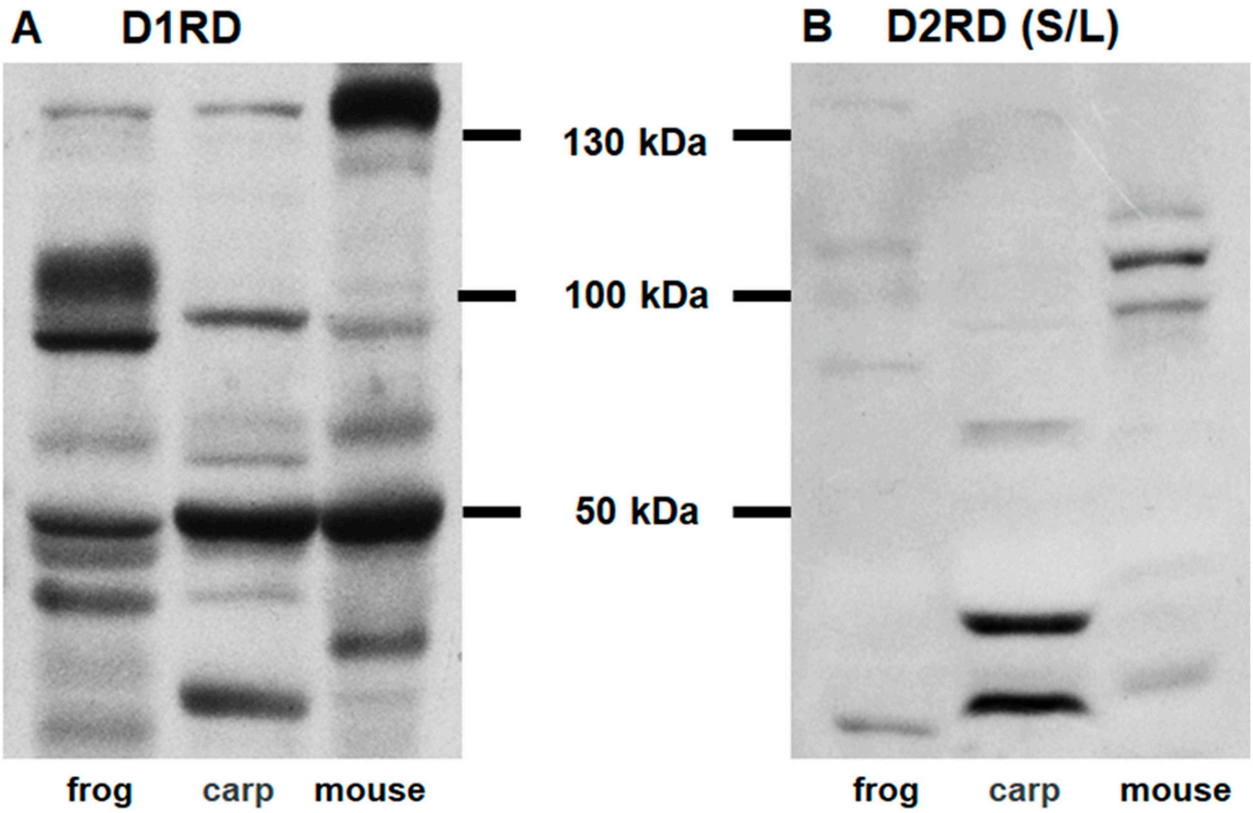

Figure S9. Figure S8: Western blotting results demonstrate the presence of immunopositive bands in the retinas of frog and carassius: (A) - D1RD-immunopositive bands in regions around 50, 100 and 130 kDa; (B) - D2RD-immunopositive bands in an area near 100 kDa. The mouse retina was used as a positive control.

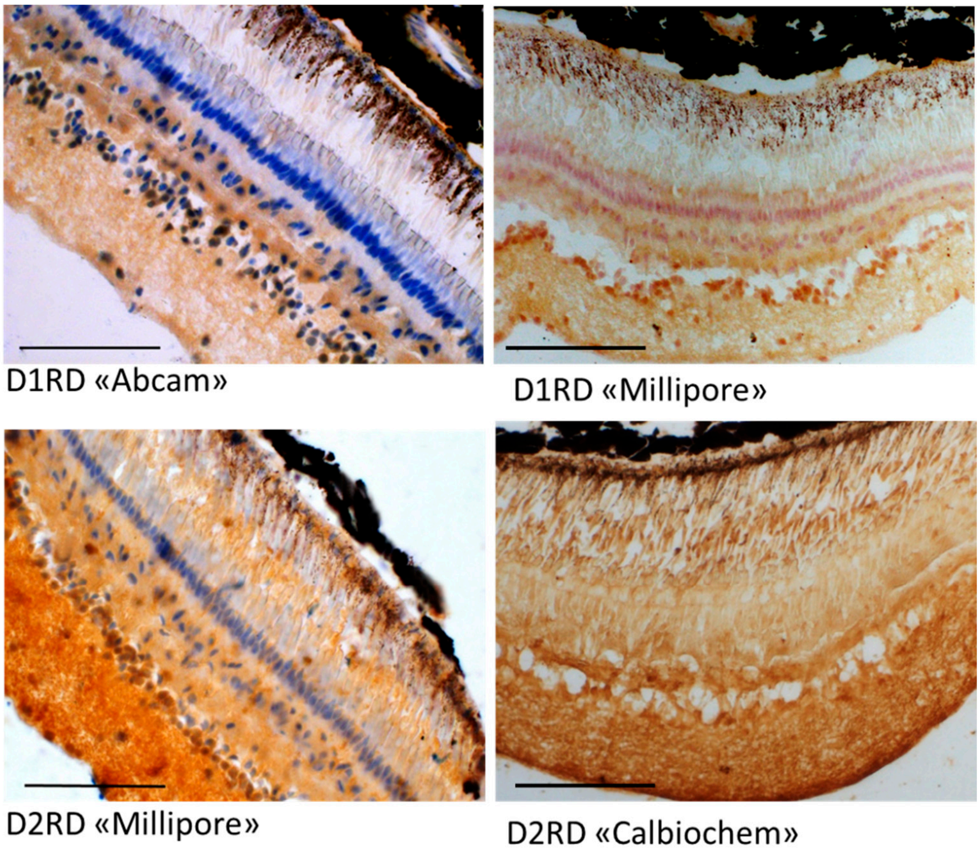

Figure S10. Immunohistochemical reactions to D1RD and D2RD in lamprey retina. Scale bars - 100 μm. The primary antibodies rabbit anti-D1RD (Abcam, 1:300), mouse anti-D1RD (Millipore, 1:200), rabbit anti-D2RD (Millipore, 1:200), rabbit anti-D2RD (Calbiochem, 1:1000), the corresponding secondary biotin-conjugated IgG anti-rabbit or anti-mouse (Vector Laboratories, Inc., 1:600), streptavidin-peroxidase solution (Sigma, 1:1000) and a solution of 0.05% diaminobenzidine (Sigma) were used for the reactions. The protocol is described in the section *Materials and methods* (*Immunohistochemistry*).
